# Quantification of cell cycle kinetics by EdU (5-ethynyl-2′-deoxyuridine)-coupled-fluorescence-intensity analysis

**DOI:** 10.18632/oncotarget.17121

**Published:** 2017-04-15

**Authors:** Pedro D. Pereira, Ana Serra-Caetano, Marisa Cabrita, Evguenia Bekman, José Braga, José Rino, Renè Santus, Paulo L. Filipe, Ana E. Sousa, João A. Ferreira

**Affiliations:** ^1^ Instituto de Medicina Molecular, Faculdade Medicina da Universidade de Lisboa, 1649-028 Lisboa, Portugal; ^2^ Kennedy Institute of Rheumatology, University of Oxford, OX3 7FY Oxford, United Kingdom; ^3^ Muséum National d´Histoire Naturelle, Département RDDM, 75231 Paris, France

**Keywords:** cell cycle, EdU, S phase, DNA replication

## Abstract

We propose a novel single-deoxynucleoside-based assay that is easy to perform and provides accurate values for the absolute length (in units of time) of each of the cell cycle stages (G1, S and G2/M). This flow-cytometric assay takes advantage of the excellent stoichiometric properties of azide-fluorochrome detection of DNA substituted with 5-ethynyl-2′-deoxyuridine (EdU). We show that by pulsing cells with EdU for incremental periods of time maximal EdU-coupled fluorescence is reached when pulsing times match the length of S phase. These pulsing times, allowing labelling for a full S phase of a fraction of cells in asynchronous populations, provide accurate values for the absolute length of S phase. We characterized additional, lower intensity signals that allowed quantification of the absolute durations of G1 and G2 phases.

Importantly, using this novel assay data on the lengths of G1, S and G2/M phases are obtained in parallel. Therefore, these parameters can be estimated within a time frame that is shorter than a full cell cycle. This method, which we designate as EdU-Coupled Fluorescence Intensity (E-CFI) analysis, was successfully applied to cell types with distinctive cell cycle features and shows excellent agreement with established methodologies for analysis of cell cycle kinetics.

## INTRODUCTION

The rate at which mammalian cells entry and progress through the different stages of their cell cycle is subject to strict regulatory mechanisms to avoid abnormal cell growth and division that may pose a threat to structure and function at the tissue level [1]. Intensive efforts have been done to accurately monitor cell cycle progression in order to better understand and predict tumor development [2]. By identifying changes in proliferation rates in response to treatment important contributes can be made to the development of anti-cancer therapeutic agents targeting specific steps of the cell cycle and the tailoring of treatment strategies for oncologic patients. In addition, determining cell cycle kinetics for distinct cell cycle stages is an important step for characterization of cancer cell lines [3].

Kinetics of S phase, in particular, can provide important information on control mechanisms and shifts in DNA replication. For instance, during early embryogenesis changes in S phase duration are frequent and reflect a progressive slowing down of firing rates of replication origins [4], while neuronal progenitor cells seem to shorten their S phase as they switch transcription factors on the path to neuron differentiation [5].

Currently, various techniques are available to estimate the duration of specific cycle phases, each with particular advantages and short-comings. Possibly the quickest, easiest and most widely used approach is to stain cellular DNA with a fluorescent dye to measure the DNA content of a cell population using flow cytometry analysis. With the aid of statistical algorithms implemented within the analysis software this results in the distribution of cells along the G1/G0 (2n), G2/M (4n) and S (2n to 4n) phases of the cycle [6, 7]. This method, however, only provides cell cycle distributions – i.e. relative lengths - at a fixed time point and suffers from variability associated with technical artifacts introduced by sample preparation, and density and condition of cells that can interfere with a uniform staining of cellular DNA [8]. Furthermore, the use of different statistical algorithms potentially introduces additional variability in the interpretation of DNA measurements between laboratories [9, 10].

Higher sensitivity strategies providing data on absolute durations of each stage of the cell cycle usually involve incorporation of detectable nucleoside analogues, the most widely used being the thymidine analogue BrdU (5-bromo-2′-deoxyuridine). BrdU is incorporated into cellular DNA during replication to tag cells in S phase, allowing their identification by immunofluorescence microscopy or flow cytometry [11]. BrdU has become standard use in proliferation studies for the past two decades as it significantly reduced the cost and time associated with previously used radioactive analogues (e.g. tritium-labelled thymidine). A drawback, however, is that antibody-based detection of BrdU has poor stoichiometry and requires a DNA denaturation step. This step, essential to expose incorporated BrdU to antibodies, can induce degradation of DNA structure and cause variability in the detected fluorescent signals [12].

In one immunofluorescence microscopy-based approach cell populations are briefly pulsed with BrdU to mark cells traversing S phase, and subsequently checked in mitosis over incremental chasing periods. Parameters on cell cycle phases can then be estimated from the time required for BrdU-labelled cells to reach M phase, yielding absolute G2 duration, and from the time BrdU-positive cells persist showing up in mitosis, corresponding to absolute S phase duration [13, 14]. This method boasts high resolution and reproducibility, although the technical steps involved in sample preparation and microscopic analysis can be very time consuming. Instead of screening for tagged mitotic fractions to identify cells that have left S phase other options involve pulsing replicating cells with two distinct nucleoside analogues at different times; or else, synchronizing the entire cell population to ensure an homogeneous entry in S phase and removal of the noise associated with double-pulsing methods [15]. Dual labelling requires the simultaneous use and detection of two antibodies specific for different analogues, hence special care needs to be taken to avoid cross hybridization signals [16]. Cell synchronization, on the other hand, carries the risk of disturbing normal cycle progression and inducing cell death, even when performed avoiding the use of drugs that target the cell cycle [17].

In recent years another thymidine analogue, EdU (5-ethynyl-2′-deoxyuridine), has become established as a viable alternative to BrdU for labeling replicating DNA. EdU harbours a terminal alkyne group that can be detected by its highly specific covalent reaction with a fluorochrome-conjugated azide. This property confers several advantages over BrdU, namely extremely high sensitivity and ease of use, along with the small size and high intracellular penetration capability of EdU reagents (1/500^th^ the size of an antibody molecule). This eliminates the need for the harsh cell permeabilization and DNA denaturation steps typical of antibody-based detection techniques [12, 18]. The characteristics of the EdU-azide reaction further suggest the potential for optimum stoichiometry detection of EdU incorporated into DNA by a quantitative methodology such as flow cytometry.

We therefore reasoned that, instead of just scoring fractions of EdU-positive cells, it would be possible to extract accurate information on the kinetics of S phase by measuring the fluorescent intensities stemming from EdU-substituted DNA (EdU-DNA). The basic assumption was that, by pulsing asynchronous cell populations with EdU for incremental periods of time, when pulsing times match the length of S phase at least a cohort of cells would be labelled for a full S phase. These cells should thus show maximum labelling intensity, and the corresponding pulsing time should equal the absolute length of S phase. Further increments in pulsing times should only increase the percentage of cells featuring such intensities.

Herein, we provide compelling evidence that this principle can be applied to measure the length of S phase with high temporal resolution even under conditions where cell cycle progression is perturbed. Furthermore, analysis of the fluorescence intensity plots obtained by flow cytometry also yields additional useful information on the lengths of G1 and G2 phases of the cell cycle. This novel method, designated here as EdU-Coupled Fluorescence Intensity (E-CFI) analysis, can be used to characterize cell types featuring highly distinct cell cycle characteristics.

## RESULTS

### Effects of EdU on DNA damage response, genomic instability and cell cycle progression

Replacement of natural thymidine by halogenated or alkylated analogues, including EdU, has been shown to introduce conformational changes in the DNA helix and nucleotide pool imbalance; also, alterations in DNA synthesis and cell cycle progression, DNA damage and genomic instability, and increased cell death [19]. We have, therefore, tested the potentially noxious effects of EdU on HCT-116 cells to establish temporal and dosage constraints to the use of EdU in estimating cell cycle parameters.

**Figure 1:**
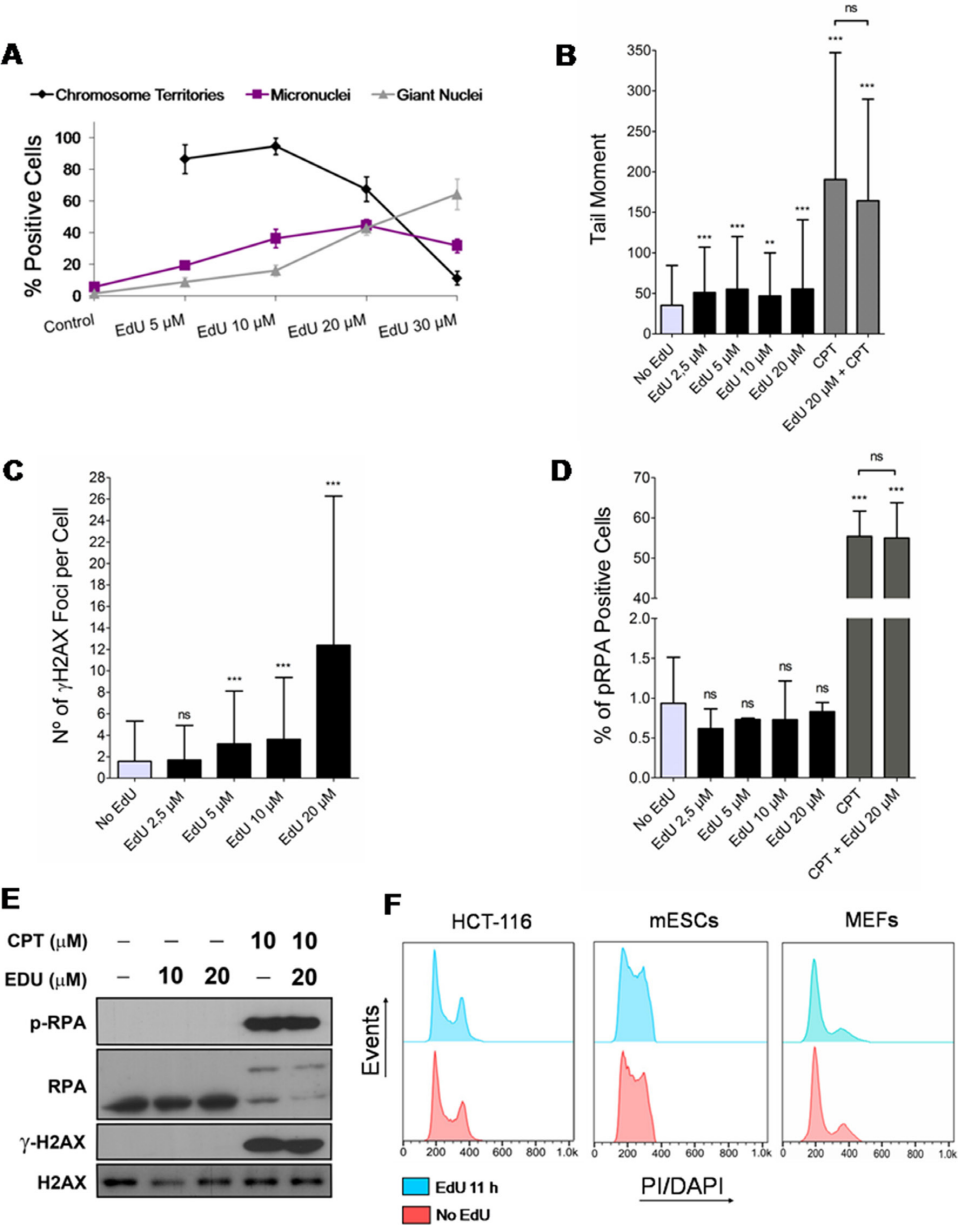
Effects of EdU on genomic instability, DNA damage and cell cycle progression (**A**) Synchronized HCT-116 cells exposed for a single full S phase (7 h) to different concentrations of EdU (5, 10, 20 and 30 μM; controls exposed to solvent/DMSO alone) and analyzed 5 days later for the presence of chromosome territories, micronuclei and giant nuclei. Note that chromosome territories cannot be formally assessed in controls not exposed to EdU. (**B**) HCT-116 cells exposed for 11 h to EdU (2.5, 5, 10 and 20 μM; negative controls exposed to DMSO) or CPT (positive control; +/– EdU 20 μM) and analyzed by alkaline single-cell gel electrophoresis (comet assay; parameter: tail moment). (**C**) HCT-116 exposed to EdU (11 h) as above and analyzed for the presence of γH2AX nuclear foci by immunofluorescence. (**D**) HCT-116 exposed to EdU (11 h) or CPT (+/– EdU 20 μM) as above and analyzed by immunofluorescence for the presence of nuclear foci concentrating RPA. (**E**) Western blots of cells exposed to EdU (11 h; +/- CPT; negative controls exposed to DMSO) and probed for phospho-RPA, RPA, γH2AX and H2AX. H2AX provides loading controls. (**F**) Flow cytometry histograms of HCT-116 cells and mESCs (PI staining), and MEFs (DAPI staining) either exposed to EdU for 11 h (HCT-116: 10 μM; mESCs: 2.5 μM; MEFs: 5 μM; blue) or not (controls; red). Results for all figures are presented as mean + SD; ***p < 0.001; **p < 0.01; ns: *p* > 0.05.

To this end, HCT-116 cells were synchronized at the G1/S transition by a double thymidine block and exposed to a range of EdU concentrations (5, 10, 20 and 30 μM) for a full S phase (see materials and methods). Cells were then analyzed 5 days later for the presence of EdU-labeled individual chromosome territories (CTs), only present in cells that underwent several rounds of mitotic division [20, 21], and of micronuclei and giant nuclei, hallmarks of genomic instability [22]. Of note, nuclei displaying EdU-labeled CTs, giant nuclei and micronuclei may concur within the same cell. At low EdU concentrations (5 and 10 μM), a significant fraction (> 80%) of labeled nuclei shows individual CTs consistent with continued cell division (Figure 1A). However, the presence of cells harboring micronuclei (19.4 ± 1.6% and 36.5 ± 5.9% for 5 and 10 μM EdU, respectively) and giant nuclei (8.8 ± 2.7% and 16.2 ± 3.5% for 5 and 10 μM EdU, respectively) were noticeably higher than in EdU-negative controls (5.9 ± 1.9% for micronuclei and 1.6 ± 0.7% for giant nuclei) (Figure 1A). At 30 μM, EdU induced a drastic reduction of CTs (only 11.3 ± 4.3% of positive cells) and a sharp increase in cells with signs of genomic instability (micronuclei: 31.9 ± 4.3%; giant nuclei: 64.3 ± 9.7%). These data indicate that in the long-term EdU induces overt signs of genomic instability.

Since the novel approach proposed here does not require long exposures to EdU, we next tested whether pulsing HCT-116 cells with EdU (2.5, 5, 10 and 20 μM) for a short period (11 h) induced DNA damage in the form of DNA breaks and replication stress; negative controls were provided by cells exposed to solvent alone. Testing the presence of DNA breaks (single- and double-stranded) by alkaline single-cell gel electrophoresis (comet assay) revealed that EdU induced statistically significant, though modest (tail moments less than twice background), levels of DNA breaks (Figure 1B, also cf. Supplementary Figure 1 in Supplementary Data for percentage of DNA in comet tails). By contrast, camptothecin (CPT; 5 μM), a known inducer of DNA breaks, and CPT plus EdU (20 μM) induced more significant amounts of DNA breakage, as expected. To specifically check for the presence of EdU-induced DNA double-stranded breaks (DSBs), HCT-116 cells were immunostained for histone γH2AX (variant histone H2AX phosphorylated on serine 139), known to accumulate as nuclear foci at genomic sites harboring DSBs [23]. Enumeration of γH2AX foci showed that EdU at 20 μM induced a significant increase in damage foci (average of 12 foci per cell; Figure 1C). Although γH2AX DNA damage foci still increased significantly after a 11 h exposure to 5 and 10 μM EdU (3 foci per cell on average), this increase was only twice background levels, becoming non-significantly different from control levels at 2.5 μM (Figure 1C). Moreover, nuclear foci concentrating phospho-RPA (Replication protein A), indicative of replicative stress, were not increased in HCT-116 cells exposed to EdU 2.5, 5, 10 or 20 μM for 11 h (Figure 1D). In accordance, western blotting analysis for the presence of increased levels of phospho-RPA and γH2AX after short term exposures to EdU (11 h; 10 and 20 μM) did not show any noticeable difference relative to EdU-less controls; however, as anticipated, cells treated with CPT (plus/minus 20 μM EdU) displayed high levels of both phospho-RPA and γH2AX (Figure 1E). Importantly, exposure of different cell types namely HCT-116, mouse embryonic fibroblasts (MEFs), and mouse embryonic stem cells (mESCs) to EdU (10, 5 and 2.5 μM, respectively; 11 h) did not change cell cycle profiles obtained by flow cytometry (propidium iodide/PI and 4',6-diamidino-2-phenylindole/ DAPI staining; Figure 1F). These data are consistent with DNA damage and replication stress sensitive checkpoints not being activated within this timeframe.

Altogether, these results show that in the long-term (5 days) even low doses of EdU induce prominent signs of genomic instability and alterations in cell division, in line with previously reported genotoxic effects of EdU [19, 24]. However, short term exposures (11 to 12 h) to low concentrations of EdU (2.5 to 10 μM) can conciliate with unperturbed cell cycle progression and thus be used in subsequent analyses.

### Stoichiometry of detection of EdU-labeled DNA

**Figure 2:**
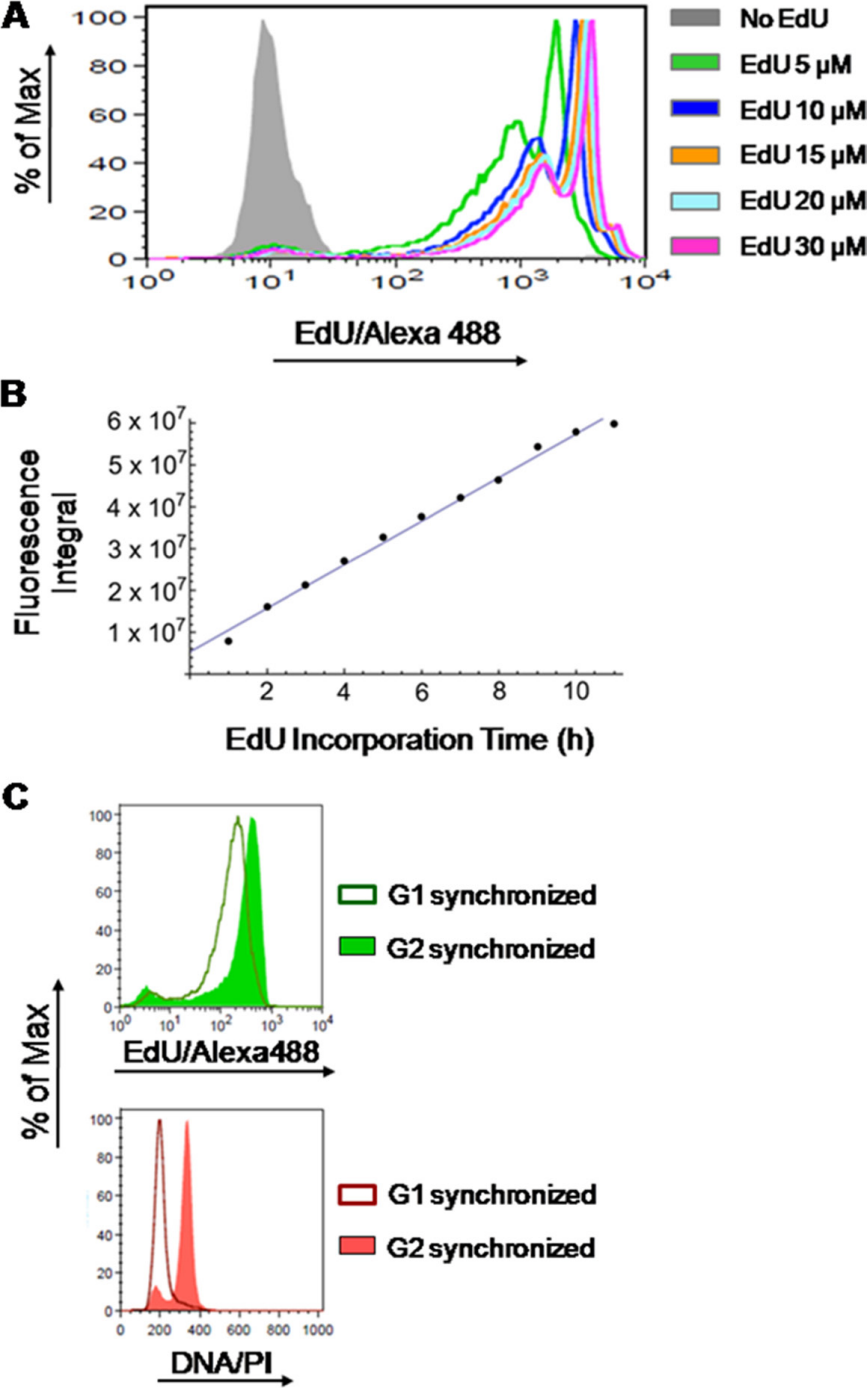
Stoichiometry of detection of EdU-labeled DNA (**A**) HCT-116 cells were exposed to different concentrations of EdU (5, 10, 15, 20 and 30 μM) or DMSO (controls) for 9 h followed by detection of EdU-DNA by Click-iT chemistry (Alexa Fluor 488). (**B**) HCT-116 cells exposed to a fixed concentration of EdU (10 μM) for incremental periods of time (1 to 11 h; 1 h increments) before detection of EdU-DNA by Click-iT chemistry. Results are expressed as an integral (sum of fluorescence intensities above background levels) for each time point. (**C**) Synchronized HCT-116 cells were allowed to incorporate EdU for a single full S phase and collected while traversing G2 stage, and later after passage into G1 stage of the next cell cycle. Histograms from cells stained for EdU-DNA (Click-iT; Alexa 488; green) and bulk DNA (PI staining; red) are depicted. Note that the EdU-coupled fluorescence peak of G1-synchronized cells (MFI: 212) has ≈48% of the intensity of the peak resulting from G2-synchronized cells (MFI: 443).

Herein, we aimed at developing a novel methodology for extracting absolute values (i.e. in units of time) on the duration of S phase through the analysis of fluorescence intensities of EdU incorporated into replicating DNA (EdU-DNA). To do so, we first assessed whether detection of EdU-DNA followed strict stoichiometry. Incorporation of different concentrations of EdU (0, 5, 10, 15, 20 and 30 μM) into cultured HCT-116 cells for a defined period of time (9 h) showed that, as expected, emitted fluorescence intensities were not proportional to EdU concentrations (Figure 2A). However, for a defined concentration of EdU (10 μM), incorporation for incremental periods of time (1 h increments) from 0 h to 11 h revealed robust stoichiometry. Indeed, increasing periods of incorporation correlated linearly with increased amounts of total fluorescence, expressed as an integral, within the cell populations (Figure 2B).

Finally, HCT-116 cells synchronized at G1/S transition by a double-thymidine block were released into S phase and allowed to incorporate EdU (10 μM) continuously for 7 h to achieve full-S labeling before harvesting. Cells were then collected first in G2/M phase (8 h after release from thymidine) and later when emerging in G1 phase of the next cell cycle (11 h after release). As quality controls for synchronization, analysis by flow cytometry (PI staining) revealed that after release from G1/S most cells progressed with remarkable synchrony (Figure 2C). Also, more than 80% of the metaphase spreads obtained from cells incorporating EdU for 7 h after release from the G1/S block displayed fluorescent labeling of EdU-DNA across the entire length of chromosome arms; this is consistent with full S labeling. In contrast, a partial (banded) EdU staining pattern was seen when cells were only briefly pulsed with EdU (10 min, 15 μM) at 2.5 h or 4 h post release from thymidine (Supplementary Figure 2 in Supplementary Data). We then compared the intensities of EdU-coupled fluorescence between cells labeled for a full S phase and collected at G2/M stages (DNA = 4n) with those allowed to progress into G1 stage (DNA = 2n). This revealed that appearance of G1 cells harboring half the amount of EdU-DNA coincided with the emergence of a half-intensity peak (mean fluorescence intensity (MFI) of the G2/M peak and the G1 peak are, respectively, 443 and 212; Figure 2C).

In all, these data showed a strict correspondence between amounts of EdU-substituted DNA and intensities of EdU-coupled fluorescence and predicated our subsequent use of EdU in experiments aimed at estimating accurate cell cycle parameters.

### Analysis of EdU-coupled fluorescence intensities

**Figure 3:**
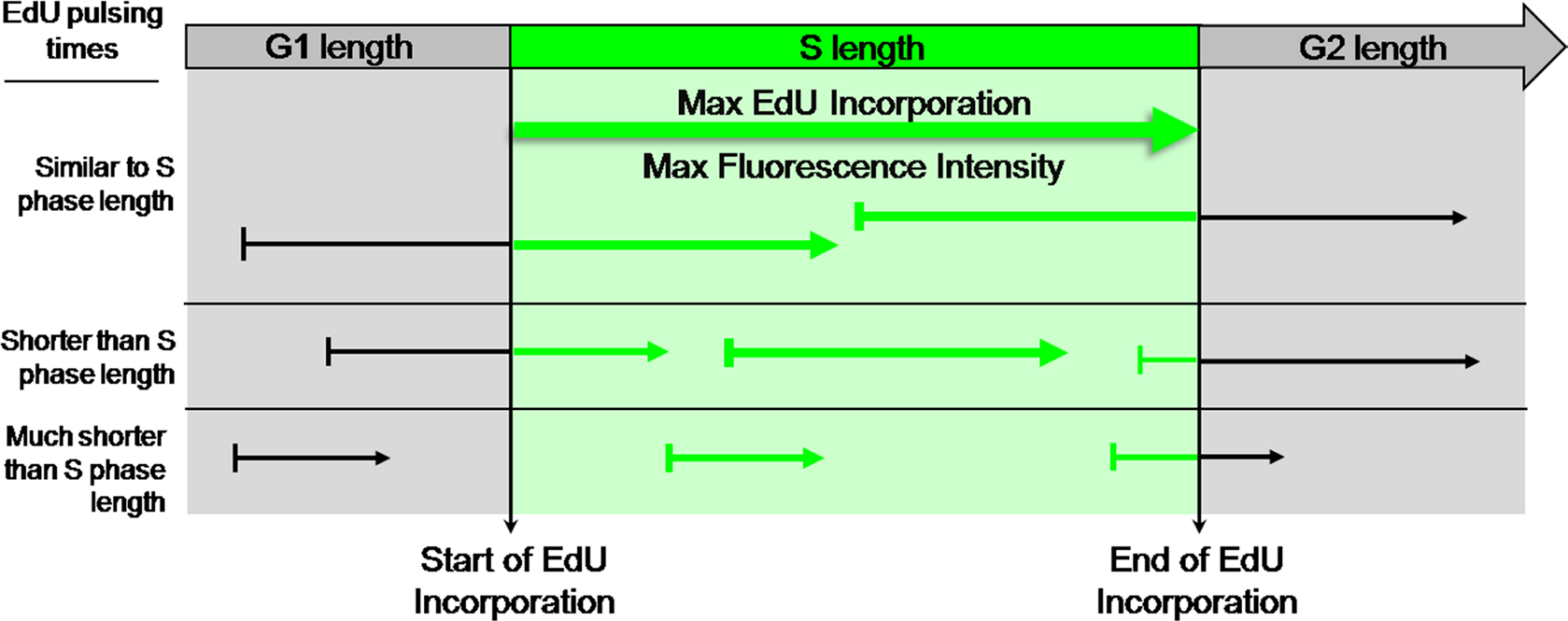
EdU-coupled Fluorescence Intensity analysis - the principle Arrows represent pulsing times of different lengths with EdU. Fluorescence intensities are denoted by thickness of arrows. Note that cells in asynchronous populations are placed at any of many possible positions of the cell cycle upon exposure to the EdU pulse. Duration of S phase is probed by pulsing cells with EdU for defined, incremental periods of time. When pulsing times match the duration of S phase the cohort of cells that, by chance, at the beginning of the pulse are initiating S phase will incorporate EdU for a full S phase. This cell population shall thus feature maximal EdU-coupled fluorescence intensity. Increasing pulsing times beyond the duration of S phase shall not increase maximal fluorescence intensities but just the percentage of cells displaying intensity maxima. Duration of S phase is thus estimated from the minimal pulsing time with EdU that elicits emergence of a population featuring maximal fluorescence intensity. Clearly, pulsing times shorter than S phase length shall not allow reaching maximal intensities. In this assay the time variable is introduced by pulsing times of defined duration and the key parameter is fluorescence intensity, not the fraction of labeled cells.

In the approach proposed here it is assumed that exposing asynchronously growing cell populations to EdU for incremental periods of time the maximum labeling intensity of EdU-DNA should be reached when the pulsing times approach, or equal, the duration of S phase. For such pulsing times, the cohort of cells in which the beginning of the pulse coincides with initiation of S phase shall become labeled for a full S phase and shall thus feature maximal labeling intensity. Absolute length of S phase shall then be equivalent to the minimum pulsing period with EdU that is required to achieve maximal EdU-coupled fluorescence intensity. Thereafter, increments in pulsing periods are expected to just increase the fraction of cells showing maximal labeling (Figure 3).

**Figure 4:**
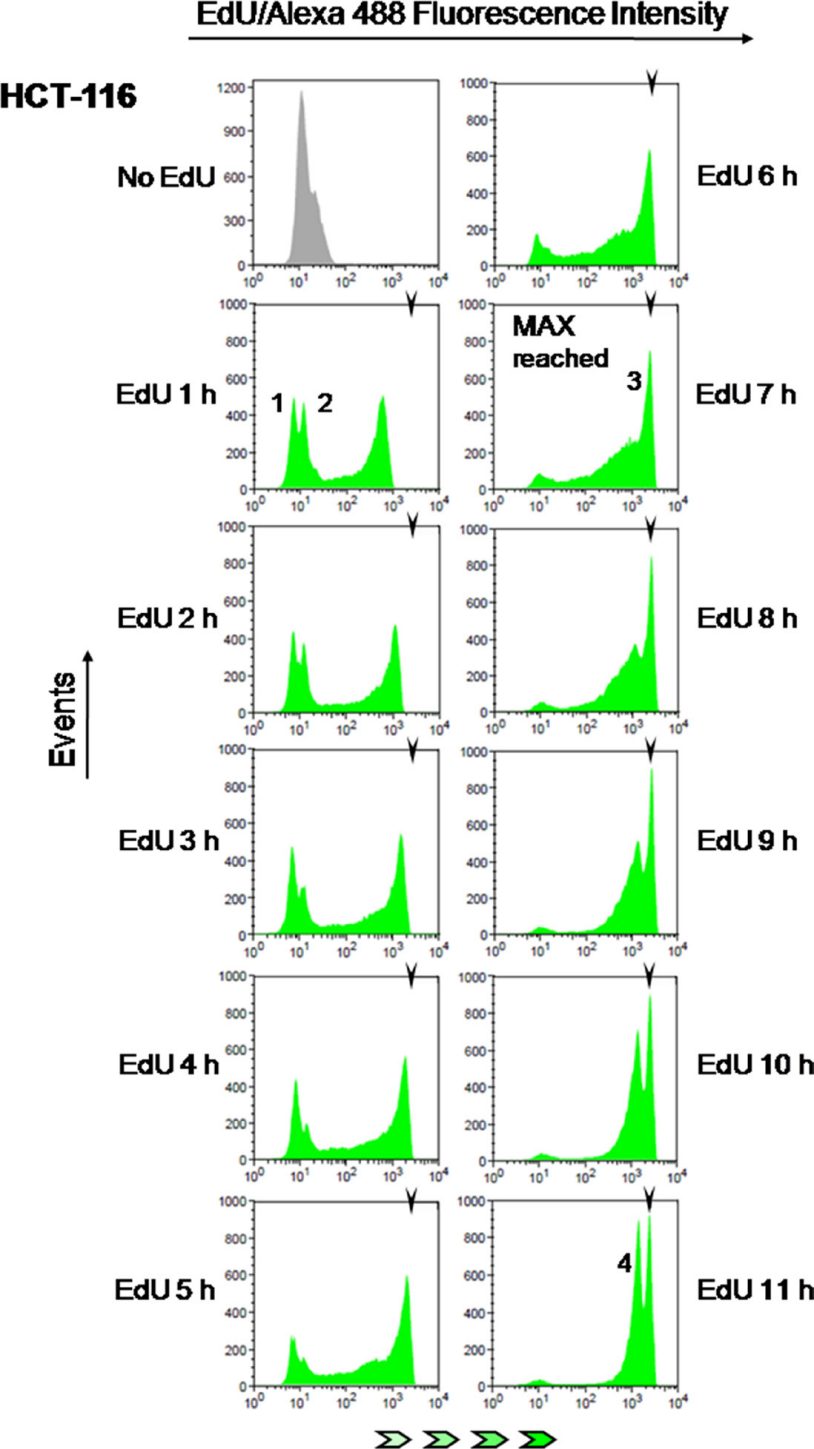
Estimation of S phase duration HCT-116 cells were exposed to EdU (10 μM) for 1 to 11 h (1 h increments) followed by detection of EdU-DNA by Click-iT chemistry (Alexa 488) and analysis by flow cytometry. EdU-coupled fluorescence intensities are displayed along the x axis in logarithmic scale. Arrowheads denote the mean fluorescence intensity (MFI) reached by maximally labeled cell populations that incorporated EdU for a full S phase (peak 3; MFI: 2677). Background peaks 1 and 2 correspond to, respectively, G1 and G2 cells that have not yet incorporated EdU. Note that peak 4 stemming from maximally labeled cells that reached G1 stage of the subsequent cell cycle features ≈1/2 the mean intensity of peak 3 (MFI: 1430, ≈53% of peak 3). Histograms are representative of one out of twelve independent experiments. Minimum number of analyzed events was 30000 per time point.

To test this idea, parallel cultures of colon cancer cells (HCT-116) were pulsed with EdU for incremental periods from 1 h to 11 h (1 h increments). Fluorescent detection of EdU-DNA was performed utilizing an azide-coupled fluorophore (Alexa 488) as part of Click-iT chemistry (cf. Materials and Methods) and bulk DNA was stained with either PI or DAPI. These experiments showed that fluorescence intensities associated with EdU-DNA increase steadily with increasing pulsing times (Figure 4; x axis represents fluorescence intensities). Maximal fluorescence intensities were first reached between 6 h and 7 h of continuous incorporation of EdU (Figure 4, 7 h time point, peak 3; MFI: 2677). According to our hypothesis this should be consistent with S phase duration of 6–7 h, indeed in good agreement with data obtained for HCT-116 cells using established methods of cell cycle analysis (cf. Table 1). To estimate the duration of S phase by E-CFI with higher temporal resolution (*n* = 10) HCT-116 cells were exposed for 6 to 8 h to EdU (10 μM) using pulsing increments of 30 min (i.e., 6, 6.5, 7, 7.5 and 8 h). This provided a more refined appraisal for S phase length (6.80 ± 0.35 h; Table 1). As expected, longer pulses with 10 μM EdU (8 h to 11 h) resulted in no discernible increment in maximal fluorescence intensities (Figure 4). However, these longer pulsing times led to an increase in the height, i.e. number of events/EdU-labeled cells (y axis), of the peak corresponding to the maximally labeled cell population (peak 3 in Figure 4). This is also anticipated given the higher chance for maximal (full S) labeling by increasing pulsing periods with EdU (Figure 3).

**Table 1:** Comparison of estimates for cell cycle phase length obtained for HCT-116 DNA-PK WT and HCT-116 DNA-PK KO through different methodologies

HCT-116 DNA-PK WT					
Method	G1	G2	G1 + G2	S	Tc
EdU-coupled Fluorescence Intensity (E-CFI)*	5.4 ± 0.95 h	3.8 ± 0.45 h		6.8 ± 0.35 h	16.00 ± 1.04 h
Fraction of Labeled Mitotic cells (FLM)		4.39 ± 0.01 h		6.75 ± 0.03 h	
Cumulative Labeling			≈7.5 h (EdU^+^ cells: 97.6 ± 1.8%)		
Leaving Fraction				6.3 ± 0.4 h	
EdU pulse-chase		5.4 ± 0.55 h		6.2 ± 1.3 h	
DNA Content (Dean Jett Fox cell cycle algorithm)**	4.8 ± 0.7 h	3.9 ± 0.6 h		5 ± 1.2 h	
DNA Content (Watson Pragmatic cell cycle algorithm)**	4.3 ± 0.6 h	4.1 ± 2.5 h		6.7 ± 2 h	

HCT-116 DNA-PK KO					
Method	G1	G2	G1+G2	S	Tc
EdU-coupled Fluorescence Intensity (E-CFI)*	6.0 ± 1.45 h	3.6 ± 0.55 h		6.75 ± 0.42 h	16.35 ± 1.54 h
Fraction of Labeled Mitotic cells (FLM)		4.4 ± 0.03 h		6.76 ± 0.02 h	
Cumulative Labeling			≈9 h (EdU^+^ cells: 94.6 ± 2.8%)		
EdU pulse-chase		4.75 ± 0.5 h		7.25 ± 0.5 h	

* Tc (Total length of cell cycle) was estimated by standard error propagation; duration of S phase was estimated as the first pulsing time with EdU after which maximal EdU-coupled fluorescence intensities clustered within 2 SDs from each other.

** Estimated cell phase durations are derived from the percentage of cells in each cell cycle stage calculated using cell cycle analysis algorithms within FlowJo software, assuming a total cell cycle length of 15 h.

We then assessed whether the minimum pulsing time with EdU required for achieving maximal fluorescence intensity of EdU-DNA, assumed here to correspond to S phase length, indeed corresponds to incorporation of EdU for a single, full S phase. To do so, exposure to EdU was restricted to a single S phase by blocking cell cycle progression in G2 stage with the Cdk1 inhibitor RO-3306. Asynchronous HCT-116 cultures were thus exposed simultaneously to EdU (10 μM) and to RO-3306 (10 μM) for 5, 7, 9 and 16 h. Controls were provided by parallel cultures exposed to EdU alone for identical periods of time and by cells not exposed to EdU (solvent alone). This experimental design ensures that a substantial fraction of cells (≈24%), i.e. those that were traversing G1 stage upon addition of EdU, will incorporate EdU for a full (and single) S phase and will not progress into the next cell cycle.

**Figure 5:**
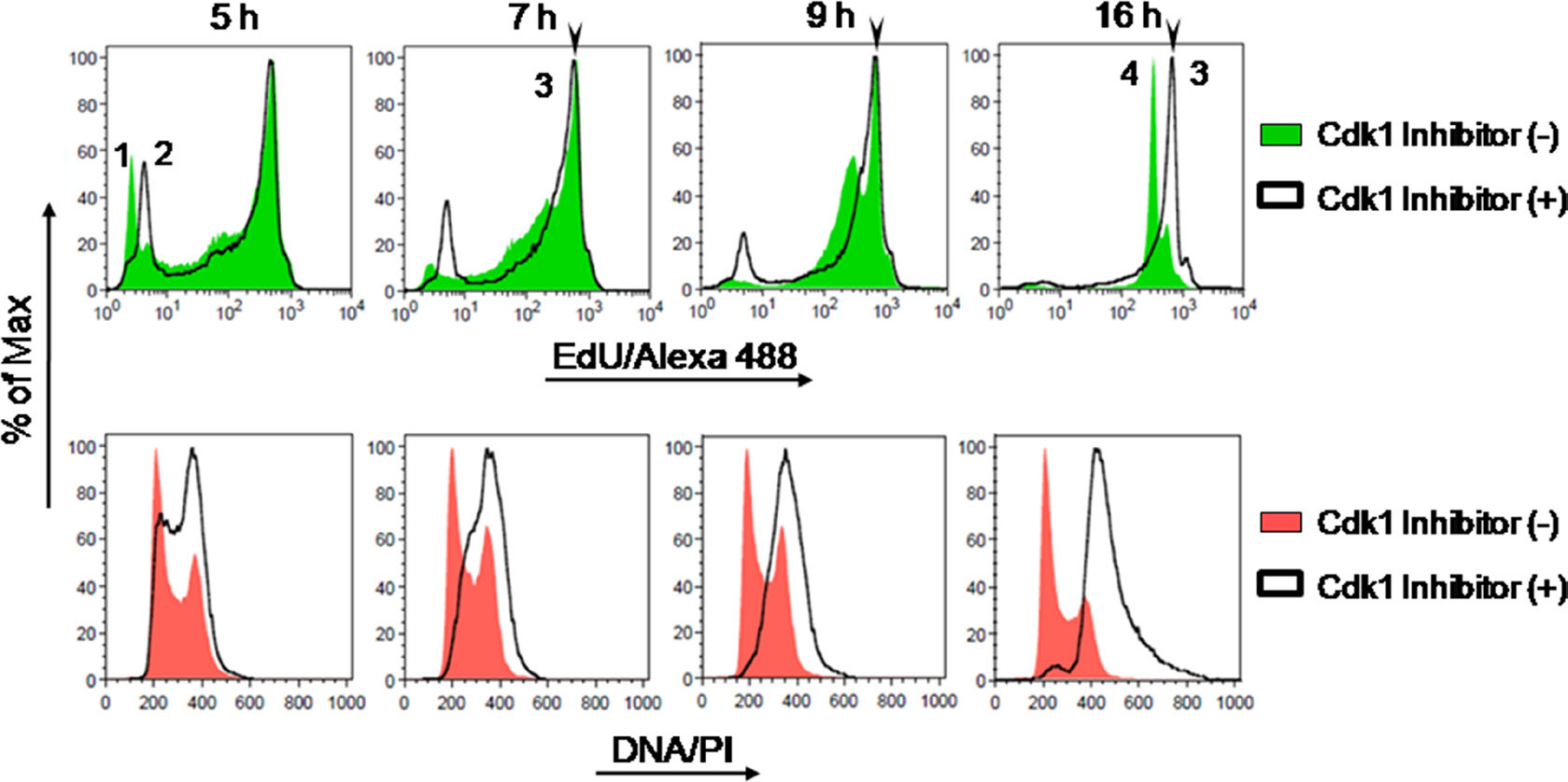
Intensity maxima of EdU-coupled fluorescence correspond to labeling for a single full S phase Asynchronous HCT-116 cells pulsed with EdU (10 μM) +/– Cdk inhibitor (RO-3306; 10 μM) and collected at defined time points (5, 7, 9 and 16 h) were analyzed by flow cytometry. Detection of EdU-DNA was done with Click-iT chemistry (Alexa 488; green) and total DNA was stained with PI (red). Fluorescence peak 1 corresponds to G1 background; peak 2 to G2 background; peak 3 to maximal intensity coupled to cells maximally labeled for EdU-DNA (MFI: 671); and peak 4 to half-maximal intensity associated to cells that reached G1 stage of the next cell cycle (RO-3306-minus group, MFI: 345). Note that maximal fluorescence intensities are similar irrespectively of the presence of RO-3306, and that peak 4 is absent from cells arrested in G2 by RO-3306.

As seen in the cell cycle histograms for bulk DNA staining (PI), after addition of the Cdk1 inhibitor the cell population initially in G1 stage progressively disappears before cells finally arrest in G2 stage, as expected (Figure 5). Analysis of EdU-coupled fluorescence further showed that maximal fluorescence intensities of EdU-DNA overlapped irrespectively of the presence of RO-3306 (Figure 5).

These data strongly support the notion that the intensity maxima seen in our initial founder experiments indeed correspond to labeling for a full, single S phase (Figure 4). Importantly, the length of S phase estimated here by flow cytometric analyses of intensity maxima of EdU-coupled fluorescence is in excellent agreement with data obtained for HCT-116 cells utilizing other, previously validated methodologies (cf. Table 1 and text further below in this section).

### Exploiting other EdU-coupled fluorescence intensity peaks

We initially focused on a sub-maximum intensity peak that in HCT-116 cells is evident after 9 h of EdU incorporation and becomes increasingly prominent thereafter (Figure 4, peak 4). Use of the Cdk1 inhibitor RO-3306 allowed elucidation of the nature of this intensity peak. When cells are blocked in their progression into the subsequent G1 phase by RO-3306 this peak is absent (Figure 5, 9 h and 16 h, peak 4). Importantly, the mean fluorescence intensity of this accessory peak is half the intensity of the maximally (full S) labeled cell population (MFI of peak 4 and peak 3 are, correspondingly, 345 and 671). Moreover, in control cells (RO-3306-minus) that progressed unperturbed for 16 h to G1 stage of the next cell cycle, this peak became the most prominent (Figure 5, peak 4). Together, these data implicate this half-maximum intensity peak as originating from G1 cells that resulted from the mitotic division of full-S-labeled cells. Since these G1 cells harbor half the amount of EdU-DNA as their progenitors and, correspondingly, emitted half the mean fluorescence intensity, this further confirms the good stoichiometric properties of the EdU detection system.

**Figure 6:**
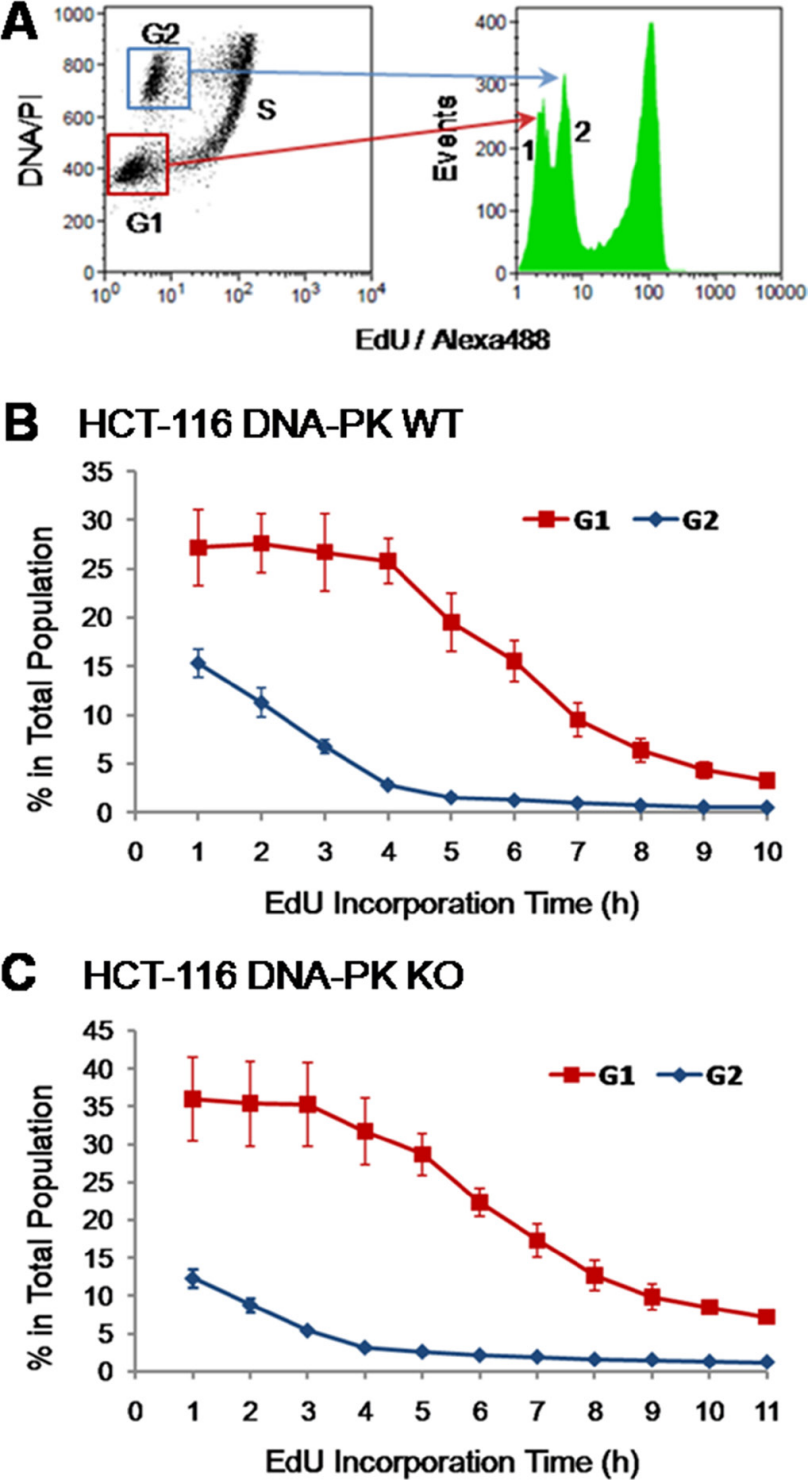
Identity of background intensity peaks (**A**) HCT-116 cells exposed for 1 h to EdU (10 μM) were stained for EdU-DNA (Click-iT; Alexa 488; green) and total DNA (PI) and subjected to dual-parameter processing (EdU *vs* total DNA). Gating for G1 and G2 populations and superimposing their fluorescence profiles with those of the entire cell population reveals the identity of the two background peaks. Note that the G1 peak (red frame) overlaps with background peak 1 and that the G2 peak (blue frame) overlaps with background peak 2. (**B**) HCT-116 cells were exposed to EdU (10 μM) for incremental pulsing times (1–10 h) according to the E-CFI protocol. The corresponding background peaks were decomposed in their constituent G1 (red line) and G2 (blue line) populations which were quantified over time. Data shown are from 5 independent experiments. (**C**) HCT-116 cells (DNA-PK KO) and the corresponding background peaks were processed as above for DNA-PK wt HCT-116 cells. Data shown are from 4 independent experiments. Results in B and C are presented as mean + SD.

Careful analysis of the EdU incorporation histograms depicted in Figure 4 reveals the consistent presence of additional, lower intensity peaks of fluorescence that change over time; of note, these peaks are already present in cells not exposed to EdU (No-EdU control; cf. Figure 4). Interestingly, the lower intensity background peaks seen in this EdU-negative population, likely due to the non-specific binding of the azide-Alexa 488 to bulk DNA, decomposed in two peaks after exposure to EdU even for short periods (Figure 4, peaks 1 and 2). Indeed, dual parameter analyses (EdU-coupled fluorescence *vs* total DNA/PI) showed that these two remaining peaks corresponded, respectively, to cells with G1 DNA content (2n; lower intensity peak) and G2 DNA content (4n; higher intensity peak) (Figure 6A). Cells with intermediate DNA contents (2n to 4n; S population), contributing to intermediate background intensities, have shifted to higher intensity regions upon incorporation of EdU leaving behind the double-peak (G1+G2) configuration of the background staining (Figure 6A, peaks 1 and 2). We note that background peaks do not always present the double-peak configuration. However, these peaks were consistently present in the many experiments performed here, acting as robust markers for the EdU-negative G1 and G2 populations. As expected, under continuous exposure to EdU these G1/G2 background peaks progressively disappear as cells initially at G1 and G2 stages move steadily into S phase and acquire strongly fluorescent EdU-coupled signals (Figure 4). We reasoned that the dynamics of these G1/G2 background peaks during time-course experiments may reflect the absolute lengths of G1 and G2 stages. The duration of G2 stage shall therefore correspond to the period of time during which cells with G2 DNA content (4n) persist featuring background staining. Since this cohort of G2 cells feeds into the next G1 phase, the duration of G1 shall be estimated after subtracting the length of G2 phase from the total duration of the G1 (2n) background peak.

We then utilized dual parameter analysis (EdU-coupled fluorescence *vs* total DNA/PI) to monitor over time the dynamics of G1 and G2 cell populations that are EdU-unlabeled, i.e. just featuring non-specific background staining. As shown in Figure 6B (*n* = 5) the percentage of G2 cells in the whole population steeply declined over time, reaching baseline levels after ≈4 h of exposure to EdU (G2 length: 3.8 ± 0.45 h; Table 1). After an initial plateau, the percentage of G1 cells decreased until 8–9 h of EdU incorporation followed by a smoother decline afterwards (Figure 6B). The initial plateau highlights the exit of G1 cells into S phase being compensated by entry into G1 stage of cells from the preceding G2 phase; the slower decline after the 8 h time point underscores the further existence of a minor population in the G1 compartment (< 5%) comprised of slow-(or non-)cycling cells. The length for G1 phase (5.40 ± 0.95 h) was estimated as the duration of G2 subtracted from the time for decline of the whole G1 population to baseline levels; this provided a good match to data gathered using validated methods (Table 1).

To further test the sensitivity of this approach we introduced in our analyses HCT-116 cells that are deficient (knock-out/KO) for the DNA repair enzyme DNA-dependent Protein Kinase (DNA-PK; HCT-116 DNA-PK KO). Using the EdU-pulsing method described herein (E-CFI) HCT-116 DNA-PK KO cells reached maximum EdU-coupled fluorescence intensity after ≈7 h of EdU incorporation (Supplementary Figure 3 in Supplementary Data). As previously performed for HCT-116 (DNA-PK wt) cells, short (30 min) increments in EdU pulsing between 6 and 8 h allowed a more accurate estimate for S phase length in HCT-116 DNA-PK KO cells (6.75 ± 0.42 h; *n* = 6; Table 1). This value is similar to that obtained for the DNA-PK proficient (wt) HCT-116 cells used throughout this research, and was confirmed by previously validated methodologies (Table 1).

We subsequently tested in HCT-116 DNA-PK KO cells, as described above for HCT-116 cells, whether quantitative analysis of G1/G2 background peaks again provided accurate values for the lengths of G1 and G2 phases. Analysis of five independent experiments showed that the percentage of G2 cells sharply decreased over 4 h of EdU incorporation before reaching baseline levels (Figure 6C). This is consistent with a G2 phase length (3.60 ± 0.55 h; *n* = 5) in HCT-116 DNA-PK KO cells that is similar to HCT-116 cells that are proficient for DNA-PK (Table 1). However, the decline in G1 cells lasted longer in DNA-PK KO cells than in their DNA-PK-wt counterparts. Near-baseline levels were reached at 9 h, with a slower decline thereafter (Figure 6C). After subtracting the duration of G2 this yields a length for G1 phase that is slightly higher (6.0 ± 1.45 h; *n* = 5) for DNA-PK KO than for DNA-PK wt HCT-116 cells (Table 1). Note that the fraction of slow/non-cycling cells (between 5 and 10%) is clearly more prominent than in HCT-116 cells harboring wt DNA-PK (< 5%) (Figures 6B and 6C). Indeed, the fraction of slow/non-cycling cells which do not incorporate modified deoxy-nucleosides even after prolonged exposure times was also shown to be higher in HCT-116 DNA-PK KO cells using other methods of cell cycle analysis (Figures 8C and 8D).

**Figure 7:**
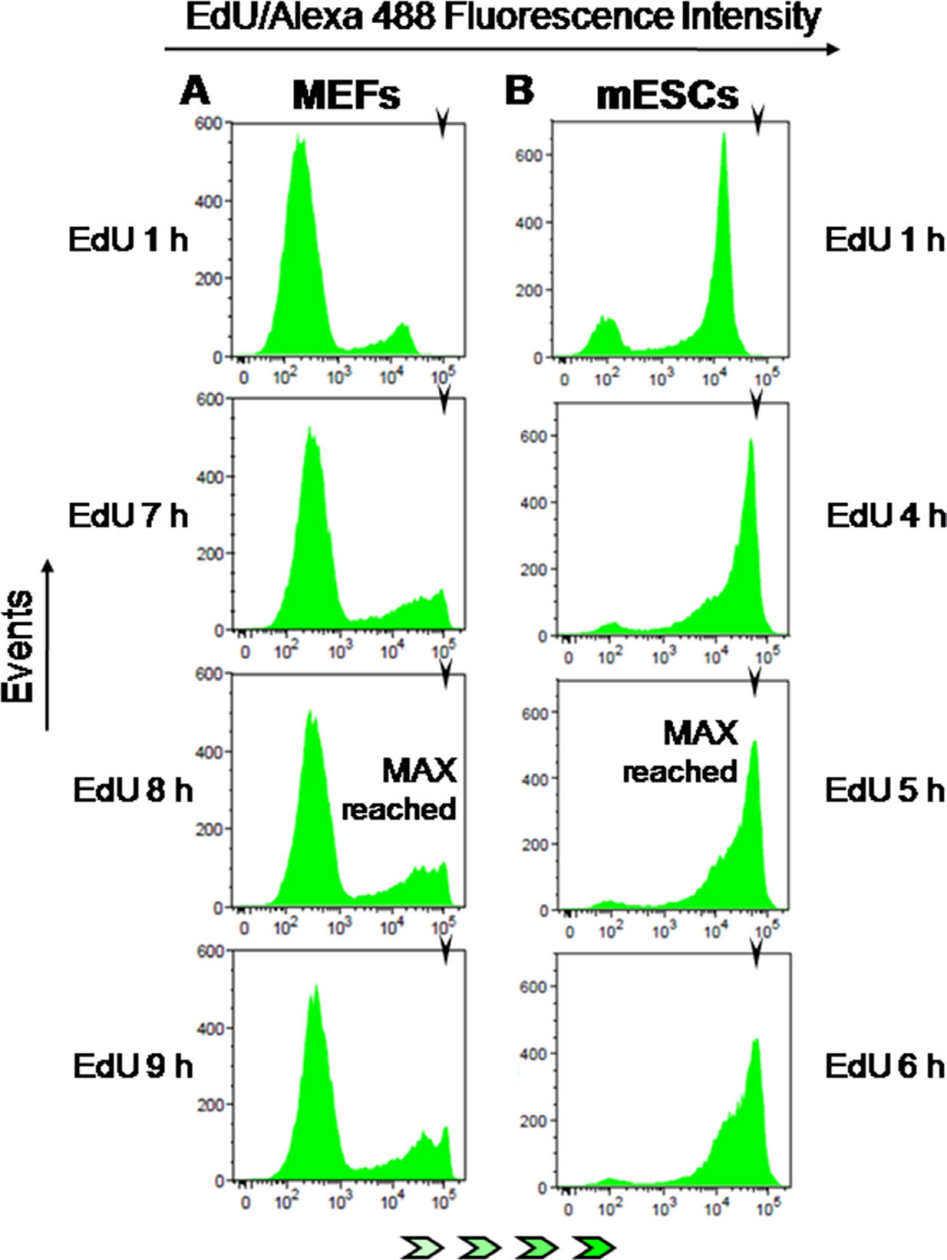
EdU-coupled fluorescence intensity analysis in non-transformed mouse cells Arrowheads denote the mean fluorescence intensity (MFI) reached by maximally labeled cell populations that incorporated EdU for a full S phase. EdU-coupled fluorescence intensities are displayed along the x axis in logarithmic scale. (**A**) MEFs were pulsed with EdU (5 μM) for 1 to 11 h (1 h increments) followed by detection of EdU-DNA by Click-iT chemistry (Alexa 488) and analysis by flow cytometry. Only select pulsing times are shown. Note that maximal fluorescence intensity is reached at 7–8 h. (**B**) Mouse ESCs were pulsed with EdU (2.5 μM) and processed as above. Maximal fluorescence intensity is reached at 5 h.

**Figure 8:**
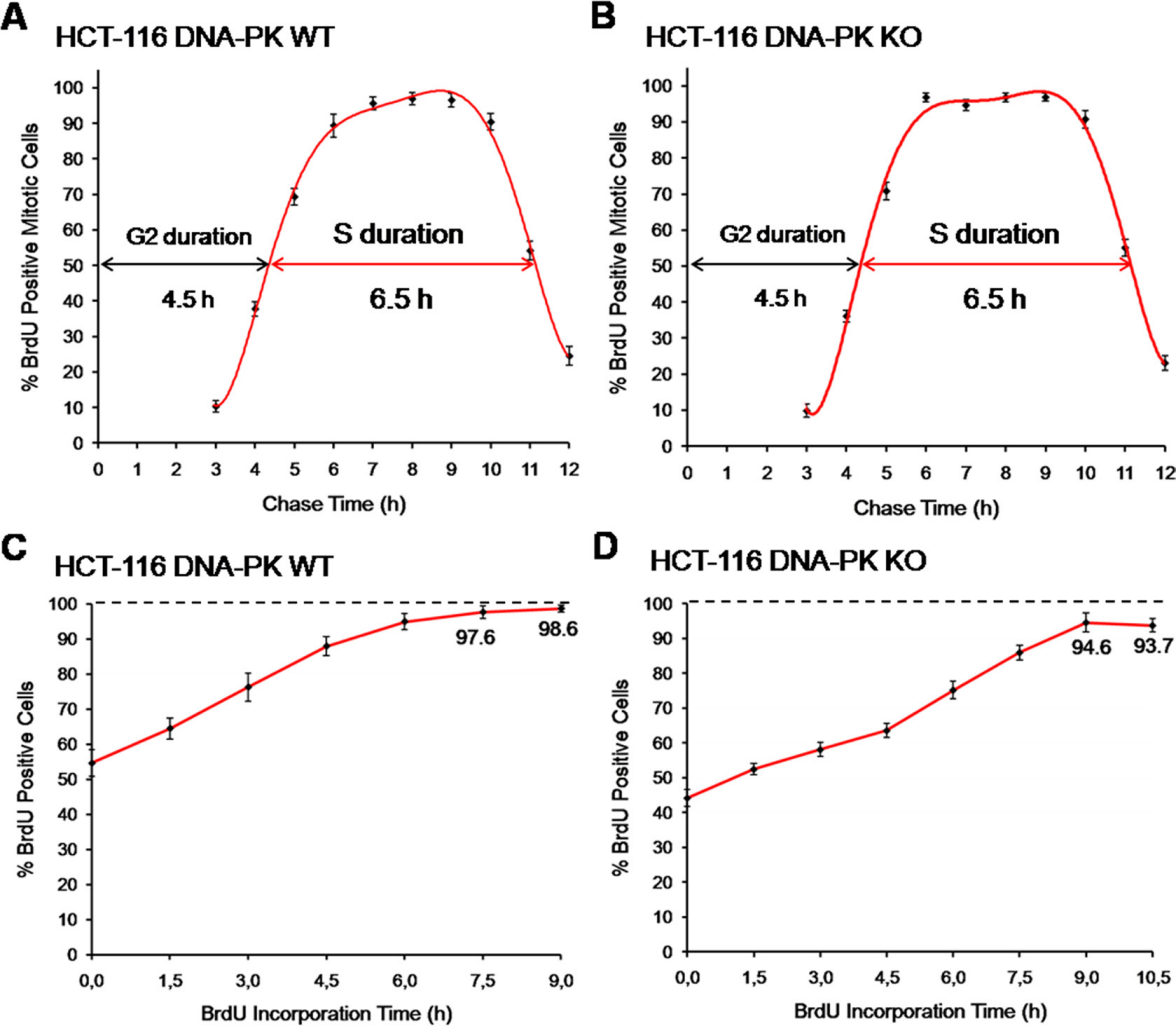
Comparison with other methods of cell cycle analysis (**A**) Fraction of labelled mitoses, HCT-116 cells. Cells were briefly pulsed with BrdU (20 μM; 15 min) and collected hourly up until 12 h after pulsing. The fraction of BrdU-positive mitotic cells was assessed per time point. Three independent experiments were performed per time point, each scored in technical triplicates; a minimum of 300 mitotic cells were counted per technical triplicate. (**B**) Fraction of labelled mitoses, HCT-116 DNA-PK KO cells. Cells were processed and scored as above for HCT-116 DNA-PK wt cells. Shown are results from three independent experiments. (**C**) Cumulative labeling, HCT-116 cells. Asynchronous HCT-116 cells were pulsed with BrdU (10 μM) for incremental periods (1.5 to 9 h; 1.5 h increments). Three independent experiments were performed per time point, each scored in technical triplicates; a minimum of 600 cells were counted per technical triplicate. (**D**) Cumulative labeling, HCT-116 DNA-PK KO cells. Cells were processed and scored exactly as in C, except that continuous pulsing with BrdU was extended to 10.5 h. Shown are results from three independent. All results are presented as mean + SD.

In sum, these data highlight the relevance of analyzing other peaks present in EdU-coupled fluorescence intensity histograms. Specifically, it was shown that quantitation of background intensity peaks provides accurate measurements for the lengths of G1 and G2 phases. These low intensity peaks also allow quantitative estimates of slow/non-cycling cells within a population.

### EdU-coupled fluorescence intensity analysis in non-transformed mouse cells

We next tested whether the analysis of fluorescence intensities associated with EdU-DNA could be applied to accurately judge cell cycle parameters in other cell types, namely in non-transformed cells. To this end, we utilized pre-quiescent (passage 26–28) mouse embryonic fibroblasts (MEFs) and mouse embryonic stem cells (mESCs). These cell types were chosen for their remarkably different duplication times. Pre-quiescent MEFs duplicate over a period of days, with a large proportion of cells in G1 and G2 stages (Figure 1F). By contrast, when under logarithmic growth mESCs feature a short cell cycle length with fast gap and S phases [25]. Given the exquisite sensitivity of ESCs to EdU [26], in this set of experiments we have consistently used lower doses of EdU (2.5 and 5 μM).

MEFs and mESCs were therefore exposed for increasing periods of time (0 to 11 h; 1 h increments) to EdU (MEFs/5 μM; mESCs/2.5 μM) before analysis of EdU-DNA fluorescence intensities by flow cytometry, as previously described. This showed that MEFs reached maximal intensity after 8 h of continuous incorporation of EdU (Figure 7A). As expected for pre-quiescent cell populations with a long G1 phase, the G1/G2 background peaks remained remarkably stable over the incremental pulsing periods used here (Figure 7A plus data not shown). By contrast, mESCs displayed maximal intensity of EdU-coupled fluorescence after just ≈5 h of exposure to EdU (Figure 7B; MFI: 60636). Also, in mESCs the G1/G2 background peak decreased to residual levels after incorporation of EdU for 4 to 5 h (Figure 7B plus data not shown). Quantitative analysis of background peaks as performed above for HCT-116 cells showed that G1 and G2 phases lasted ≈2 h each (data not shown). These data are consistent with a total length of ≈9 h for the full cell cycle in mESCs, in excellent agreement with previously published data [25].

These experiments further extend the applicability of the novel E-CFI method to other cell types, even under the constraint of utilizing very low concentrations of EdU.

### Comparison with other methods of cell cycle analysis

We subsequently tested how the method developed here compared to previously implemented assays aimed at estimating cell cycle parameters.

In a robust pulse-chase method–termed “Fraction of Labeled Mitoses” - that allows absolute estimates of the duration of S and G2 phases, cells are first briefly pulsed with a radioactive or a modified deoxy-nucleoside (e.g. BrdU), chased in mitosis for incremental periods of time and scored for the presence of labeled chromosomes [13, 14, 27]. In this assay, the time between pulsing and the emergence of the first labeled mitotic cells (≈50% of labeled cells) equals the absolute duration of G2. The time period during which the cohort of cells previously labeled in S phase with BrdU continues showing up in mitosis with BrdU-labeled chromosomes corresponds to the absolute duration of S phase [14].

Parallel cultures of HCT-116 cells (DNA-PK wt and DNA-PK KO) were therefore pulsed with BrdU (20 μM; 15 min), collected at hourly intervals up until 12 h after pulsing, immuno-stained for BrdU, and the percentage of BrdU positive mitotic cells (prometaphase plus metaphase stages) was assessed for each time point. These experiments showed values for S and G2 phases very close to those obtained using E-CFI, for both DNA-PK deficient and proficient HCT-116 cells (Figure 8A and Table 1).

We also used the “Leaving Fraction” method in which cells are first pulsed with a modified deoxy-nucleoside, chased for a defined time period in medium free of modified nucleosides, and subjected to a second pulse with a differently modified nucleoside before collection (cf. Materials and Methods). The fraction of cells labeled by the first modified nucleoside but not by the second corresponds to the so-called leaving fraction, i.e. the fraction of cells that although initially in S phase have reached G2 during the chase period. Through extrapolation, the time required for all cells to leave S phase, which equals S phase duration, can be estimated as an absolute value (see also Materials and Methods) [28–30]. Notably, this microscopy-based method also yielded an S phase duration (6.3 ± 0.4 h) similar to that we have obtained throughout this research for HCT-116 cells using E-CFI (Table 1).

In a third approach we used a “Cumulative Labeling” method, also known as “Saturation Labeling” [31], to assess the absolute duration of G1 plus G2 stages. The underlying principle is that upon a brief exposure to a modified deoxy-nucleoside this is exclusively incorporated into the replicating DNA of cells traversing S phase. However, if pulsing times are progressively extended to encompass the duration of G1 plus G2 all cells initially at these stages will ultimately be allowed to reach S phase and thus to incorporate the analogue. Therefore, the minimal pulsing times with the analogue that allow labeling of the whole cell population will match the combined duration of G1 plus G2 stages for that population [32, 33]. HCT-116 cells (DNA-PK wt and DNA-PK KO) were thus pulsed with BrdU (10 μM) for incremental periods of time up until 10.5 h before scoring by fluorescence microscopy. Again, the estimates for the combined duration of G1 plus G2 (HCT-116 DNA-PK wt: 7.5 to 9 h; HCT-116 DNA-PK KO: ≈9 h) closely agree for both cell lines with those obtained using E-CFI (Figure 8C, 8D and Table 1). Interestingly, for both cell lines a sub-population of slow-/non-cycling cells was identified that was slightly more prominent in DNA-PK KO cells, as previously seen in experiments using E-CFI (Figure 8C and 8D).

Additionally, we used a pulse-chase methodology for estimation of the lengths of S and G2 phases [19]. To this end, HCT-116 (DNA-PK wt and DNA-PK KO) and mESCs were pulsed with EdU (10 μM and 5 μM, respectively) for 30 min and collected either immediately (no chase), or else chased in EdU-free medium before collection at hourly intervals. The length of G2 phase was estimated as the period of time between the end of the EdU pulse and the time point at which the population harboring G2 DNA content (4n) showed the highest percentage of EdU-positive cells. This expectedly occurs when the cohort of EdU-labeled cells (i.e. in S phase during EdU pulsing) reaches the G2/M transition after traversal of G2 phase. The interval between this latter time point and the time point where the population with 4n DNA content reaches its lowest percentage of EdU-positive cells was considered as the duration of S phase. This corresponds to the period during which the cohort of EdU-labeled cells fully passes through the G2/M transition into the next G1 phase.

Analysis by dual-parameter flow cytometry (EdU *vs* total DNA/PI) showed that the cohorts of EdU-labeled *vs* unlabeled cells progress evenly over time between cellular compartments harboring 2n and 4n DNA amounts (HCT-116 cells; Supplementary Figure 4 in Supplementary Data, plus data not shown). However, as depicted in Supplementary Figures 4 and 5 (Supplementary Data; HCT-116 cells), for each of four independent experiments it proved difficult to judge for cells with 4n DNA the time points at which the percentage of EdU-positive cells reached a maximum. Indeed, in these bowl-shaped curves these maximal values - typically reached at 4 to 6 h after the EdU pulse - are almost identical between neighbor time points (cf. Supplementary Figure 5). When these data were combined (*n* = 4) in a single line chart this showed that the maximal percentages of EdU-positive cells (4n DNA) seen at the 4, 5 and 6 h time points were not significantly different (81.2 ± 12.3%, 86.6 ± 9.7%, 86.4 ± 3.5%; 4, 5 and 6 h, respectively; Supplementary Figure 6A in Supplementary Data). Within the constraints of this method, we estimated the length of S and G2 phases for HCT-116 cells (DNA-PK wt, *n* = 5; DNA-PK KO, *n* = 4; Table 1). These values, despite their lower temporal resolution in particular for S phase, are in broad agreement with those obtained by E-CFI (Table 1). Assessment of mESCs by EdU pulse-chasing yielded better homogeneity between different experiments as shown in the graph depicting pooled data (*n* = 4; Supplementary Figure 6B in Supplementary Data). The durations of S (5.75 ± 0.5 h) and G2 (4.25 ± 0.5 h) phases were estimated taking the 4 h time point as that corresponding to the highest percentage of EdU-positive cells within the 4n DNA population (cf. Supplementary Figure 6B in Supplementary Data).

Finally, we combined the commonly used analysis of the cell cycle by flow cytometry after PI staining of DNA with estimates of the absolute duration of the cell cycle in HCT-116 cells. This latter parameter is essential to convert the percentage of cells at a given cell cycle phase, which directly correlates with the relative length of that same phase in reference to the full cell cycle, into absolute lengths (i.e., in units of time).

Taking the duration of the cell cycle as the absolute parameter (14–15 h for HCT-116 cells), the percentage of cells seen at each cell cycle stage in flow cytometry histograms of PI-stained DNA was then converted into absolute lengths (hours). We note that the two available mathematical models within FlowJo, Watson Pragmatic (WP) and Dean/Jett/Fox (D/J/F), yielded discrepant percentages for each of the cell cycle stages in HCT-116 cells. The values obtained through the WP algorithm provided a better fit to the data gathered throughout this research using different methodologies, including E-CFI (cf. Table 1, and Supplementary Table 1 in Supplementary Data). However, using either of these algorithms (WP or D/J/F) we could not generate any reliable estimates for the much less canonical cell cycle histograms from exponentially growing mESCs (depicted in Figure 1F).

In sum, the E-CFI method described herein shows excellent concordance with data obtained through various well established methods of cell cycle analysis.

## DISCUSSION

We have herein described an assay which we termed E-CFI that allows easy and accurate measurements of the absolute length of all stages of the cell cycle (G1, S and G2/M) by flow cytometry.

The approach of reference to analyze the duration of the different stages of the cell cycle is based on flow cytometric analysis of cellular DNA stained with a fluorescent dye that binds stoichiometrically, and thus allows measurement of DNA content [34]. This provides values on the proportion of cells found at each phase (G1, S and G2/M) which directly correspond to relative durations in reference to the length of a full cell cycle. Despite the use of algorithms that attempt at fitting Gaussian curves to each phase, a clear distinction between cells traversing very early or late S phase from cells in G1 and G2 phase, respectively, remains difficult by single parameter DNA analysis [10, 34]. This difficulty becomes more obvious for cell types with atypical cell cycles namely with very long G1 phases, as is the case of pre-quiescent MEFs, or very short G1 and G2 phases, such as mESCs (Figure 1F; also Table 1). In such cases, under or overestimation of the length of S phase is the typical result [35]. Indeed, applying two different mathematical models, Dean/Jett/Fox and Watson Pragmatic, discrepant data was obtained for HCT-116 cells with D/J/F yielding unusually short durations for S phase (≈5 h; Table 1, and Supplementary Table 1 in Supplementary Data). Unfortunately, clear criteria for choice between different algorithms do not exist. In mESCs, in which S phase typically lasts more than 50% of the total cell cycle, both the D/J/F and the WP algorithms proved unreliable. Moreover, the conversion of these data on relative durations into absolute lengths for each phase requires an additional, accurate estimate of the absolute length of the cell cycle under the conditions being tested [7].

Pulse-chase methods utilizing EdU (or BrdU) also provide data on absolute durations of cell cycle phases and stand as possible contenders to the E-CFI assay described herein [19]. These methods, however, rely critically on selecting the fraction of EdU-positive cells within sub-populations of defined DNA content. They therefore share the known constraints of quantitative analyses of single parameter DNA histograms [7, 36]. As a result, estimates for the duration of S and G2 stages have lower temporal resolution (higher dispersion) than those obtained with either E-CFI or other classical, validated methods (Table 1).

Herein, we have utilized pulse-chasing with EdU on HCT-116 cells (DNA-PK wt and DNA-PK KO) and mESCs, and found that the critical time points to judge the duration of G2 and S phase proved difficult to determine unambiguously. Setting up these time points allows tracking of the cohort of EdU-positive cells over time. These correspond, specifically, to the time point(s) at which the G2 (4n DNA) population features the highest percentage of EdU-positive cells and, later, the lowest percentage of EdU-positive cells. The time interval between the end of the EdU pulse and the point(s) of maximal labeling corresponds to the length of G2; the interval between maximal and minimal labeling time point(s) corresponds to the length of S phase.

The exemplary case is provided by mESCs (Supplementary Figure 6B in Supplementary Data). In these cells, the time points corresponding to maxima of EdU-positive cells (≈92% to ≈96%) distribute broadly between 2 and 5 h after pulsing with EdU, with the minimal fraction of EdU-positive cells seen at 10 h after pulsing (cf. Supplementary Figure 6B in Supplementary Data). Taking the 2 h time point as the most significant maximum would be consistent with a ≈2 h duration of G2 phase in mESCs, in agreement with previously published data and the results obtained herein using E-CFI [25]. However, the interval between this and the 10 h time point (minimal percentage of EdU-positive cells) would lead to an excessively long estimate for S phase (≈8 h). This dilemma stems, at least in part, from the duration of G2 being much shorter than that of S phase, and also from the poor discrimination between cells at later stages of S phase (close-to-4n DNA) and genuine G2 cells. This latter issue can be appreciated at the end of the EdU pulse when most cells (> 60%) harboring 4n DNA content have indeed incorporated EdU (cf. Supplementary Figure 6B in Supplementary Data; 0 h after EdU pulse).

The assay we describe here, E-CFI, is easy to perform and allows accurate estimates of absolute lengths (in units of time) of all the different stages of the cell cycle (G1, S, and G2/M). The duration of S phase is assessed without selection of cell populations based on DNA content. Although this is required for analysis of G1 and G2 phases, these two phases can be accurately separated given the absence of cells in the intervening S phase in background peaks. The classical problem of discriminating cells at the G1/S and S/G2 borders is thus avoided [7, 36]. Furthermore, E-CFI allowed the identification and quantification of sub-populations with decreased proliferative potential (slow-/non-cycling cells) within the G1 compartment in HCT-116 cells. We note that, in contrast to E-CFI, the duration of G1 is difficult to quantify in dual-parameter histograms from EdU pulse-chase experiments (Supplementary Figure 4 in Supplementary Data).

Herein, we have used the Cdk1 inhibitor (RO-3306) to demonstrate that the intensity maxima reached after continuous incorporation of EdU correspond indeed to labeling for a single, full S phase (Figure 5). These experiments further showed that E-CFI allows accurate estimation of S phase length even when cell cycle progression is blocked. Of note, this blockage would preclude the use of other methods of cell cycle analysis namely pulse-chase, cumulative labeling and methods in which labeled mitosis are scored.

E-CFI showed excellent correlation with the highly reproducible and precise methods of “Cumulative Labeling” and “Fraction-of-labeled mitosis/FLM” [15, 31–33] (Table 1). These microscopy-based classical methods are, however, highly time consuming and thus not amenable to routine use. By contrast, E-CFI yields fast results by acting in a time-compressing fashion whereby G1, S and G2/M phases are assessed in parallel. This allows absolute estimates on the lengths of each cell cycle stage to be collected over a time period that is shorter than the duration of a full cell cycle. For example, for HCT-116 cells whose full cell cycle lasts for 15–16 h, the length of all cell cycle phases can be determined in 8–9 h.

We anticipate that E-CFI may provide a very valuable tool in the analysis of drugs targeting the cell cycle in the context of cancer chemotherapy, especially if coupled to powerful multiparametric analyses using flow cytometry or high-content imaging [7, 37, 36, 38]. Also, the basic principle that predicated the development of E-CFI may be applied in the future to quantitative fluorescence microscopy-based approaches aimed at estimating absolute, accurate cell cycle parameters.

## MATERIALS AND METHODS

### Cell culture, chemicals and antibodies

Human colorectal carcinoma HCT-116 (ATCC CCL-247) and HCT-116 knock-out for DNA-PK were obtained from the laboratory of Dr. Bert Vogelstein, Johns Hopkins School of Medicine, Baltimore, MD. HCT-116 cells were cultured in McCoy's 5A Modified medium supplemented with 10% heat inactivated foetal bovine serum (FBS), 2 mM L-glutamine, 10mM MEM non-essential amino acids, and 100 U/ml penicillin/streptomycin (all from Gibco, Thermo-Fisher Scientific, Waltham, MA, USA) and maintained at 37°C in a humidified incubator at 5% CO_2_. Mouse embryonic fibroblasts (MEFs) were cultured in Dulbecco's Modified Eagle's Medium (DMEM) supplemented with 10% ES Cell-qualified FBS (Invitrogen, Thermo-Fisher Scientific, Waltham, MA, USA), 10 mM MEM non-essential amino acids, and 0.1 mM 2-mercaptoethanol (Gibco). Mouse embryonic stem cells (mESCs) were grown at 37°C in 5% CO2, in Glasgow Modified Eagle´s Medium (GMEM, Invitrogen) supplemented with 10% FBS (ES-Cell qualified), 10 mM MEM non-essential amino acids, 1% GlutaMAX, 1 mM 2-mercaptoethanol and 2 ng/mL Recombinant Human Leukemia Inhibitory Factor (LIF; serum/LIF conditions), on gelatin-coated (0.1% v/v) dishes (Nunc, Roskilde, Denmark). Cells were passaged on alternate days at a constant plating density of ≈3 × 10^4^ cells/cm^2^.

Camptothecin, RO-3306, 5-bromo-2´-deoxyuridine (BrdU), thymidine, propidium iodide (PI), 4’,6-diamidino-2-phenylindole (DAPI) and RNase A were purchased from Sigma-Aldrich (St. Louis, MO, USA).

The following antibodies were used in this research: rabbit polyclonal to histone H2A.X (ab11175, Abcam, UK [39]), mouse monoclonal IgG1 to phospho-histone H2A.X (Ser139; clone JBW 301; Merck-Millipore, Darmstadt, Germany [40]), mouse monoclonal IgG1 to RPA32/RPA2 (clone 9H8; ab2175 Abcam, UK [41]), affinity-purified rabbit polyclonal to phospho-RPA32/RPA2 (Ser4/Ser8; Cat. A300-245A; Bethyl Laboratories, Montgomery, TX, USA [42]), mouse monoclonal antibodies to BrdU (clones BU-33 and BMC 9318; Sigma-Aldrich), affinity-purified Alexa 488-conjugated and Cy3-conjugated anti-mouse secondary antibodies (Jackson ImmunoResearch Laboratories, Sacramento, CA, USA), and peroxidase-conjugated affinity-purified goat anti-mouse IgG and goat anti-rabbit IgG (BioRad Laboratories, Hercules, CA, USA).

### EdU incorporation and detection for flow cytometry

EdU (5-ethynyl-2′-deoxyuridine), supplied with Click-iT EdU Alexa Fluor 488 Imaging Kit (#C10337, Thermo-Fisher Scientific, Waltham, MA, USA), was diluted in DMSO to a final concentration of 10 mM and kept at –20°C. Typically, EdU was added to parallel cultures growing exponentially in 30 cm^2^ petri dishes to final concentrations ranging from 2.5 to 30 μM for varying lengths of time until collection. Cells exposed to DMSO (solvent) alone served as controls. Cell pellets (approx. 5 × 10^5^ cells) were vigorously resuspended in 200 μL of ice cold 2% formaldehyde in PBS and fixed for 2 min, permeabilized by subsequent addition of 1 mL of 70% ice-cold ethanol (without removal of formaldehyde), and kept on ice for a minimum of 10 min. Cells were then washed three times in 1 mL PBS containing 0.05% Triton X-100 (PBS-Tx) before detection of EdU-substituted DNA (EdU-DNA). Detection of EdU-DNA was performed according to the Click-iT EdU Alexa Fluor 488 Imaging Kit as per manufacturer's instructions. Cell pellets were incubated in 100 μl of reaction buffer for 35 min at 37°C protected from light. Cells were subsequently washed 4 × 10 min in 1 mL PBS-Tx 0.05% under constant shaking before staining of bulk DNA with PI or DAPI.

For PI staining, cell pellets were resuspended in 300 μL of a solution comprised of 10 μg/mL PI, 192 μg/mL RNase A and 0.1% Triton X-100 in PBS and incubated for 30 min on ice, followed by a further incubation for 30 min at 37°C, protected from light. For DAPI staining, cell pellets were resuspended in a solution of 1 μg/mL of DAPI in PBS containing 0.1% TritonX-100 and incubated protected from light for 1h at 37°C. Cells were washed three times in PBS-Tx before measuring their fluorescence by flow cytometry.

### Flow cytometry instrumentation and data analysis

Samples stained for EdU, PI and DAPI were analyzed using a three laser (blue-488 nm; red-640 nm; violet-605 nm) BD LSR Fortessa flow cytometer (BD Biosciences, San Jose, CA). EdU–Alexa 488 and PI signals were measured upon excitation by the blue laser using 530/30 and 695/40 bandpass filters, respectively. DAPI signals were measured upon excitation by the violet laser with the 525/50 bandpass filter. A minimum of 30000 events were acquired per experiment in slow rate mode to avoid doublets. Sample measurements were performed with FACSDiva Software (Version 6.2, BD Biosciences, San Jose, CA, USA). Data analysis, such as mean fluorescence intensity (MFI) measurements, was performed with FlowJo Software (Ashland, OR, USA). Cell debris and aggregates were excluded from the analysis using pulse processing FSC-A *vs* FSC-H, FSC-H *vs* FSC-W, SSC-H *vs* SSC-W, and FL2-A *vs* Fl2-W when appropriate.

### Immunofluorescence staining

For immunofluorescence analysis, HCT-116 cells growing on coverslips were routinely fixed in freshly prepared 3.7% paraformaldehyde in HPEM buffer (30 mM HEPES, 65 mM Pipes, 10 mM EGTA, 2 mM MgCl_2_ (pH 6.9)) plus 0.5% Triton X-100 for 10 min at room temperature before incubation with antibodies. All washes were performed with PBS containing 0.05% Triton X-100. For detection of BrdU incorporated into replicating DNA, fixed cells were further incubated with 4N HCl for 10 min to depurinate DNA and washed four times for 10 min in Tris 50 mM (pH 8) preceding incubation with anti-BrdU antibodies. For the double labeling of BrdU and EdU immunostaining of BrdU preceded detection of EdU by the Click-iT method. Antibodies used for immunofluorescence were diluted in PBS containing fish skin gelatin (0.1%) and Triton X-100 (0.05%) as follows: anti-RPA32/RPA2 at 1/200, anti-phospho-histone H2A.X (Ser139) at 1/300, anti-BrdU at 1/100 and Cy3-conjugated anti-mouse secondary antibodies at 1/100. After immunolabeling, total DNA was stained with DAPI (0.5 μg/mL) and coverslips were mounted in Vectashield (Vector Laboratories Inc., Burlingame, CA, USA) before analysis by fluorescence microscopy.

### Confocal microscopy

Samples were examined using a Zeiss 510 confocal microscope (Carl Zeiss, Jena, Germany) equipped with lasers giving excitation lines at 405, 488 and 543 nm. Data from the channels were collected separately using narrow-band-pass filter settings. In multiple staining experiments, the laser intensities were adjusted to avoid bleedthrough between channels. Data were collected with two- to fourfold averaging at resolution of 1024 × 1024 pixels using pinhole settings between 1.05 and 1.10 airy units. Data sets were processed using Zeiss 510 version 2.8 software package and were subsequently exported for preparation for printing using Adobe Photoshop, version CS5.1.

### Other methods for estimation of cell cycle parameters

In the methods described below BrdU was administered either as a single pulse or in association with a second, distinct pulse with EdU (double-pulsing). Asynchronous cultures of HCT-116 cells were grown on glass coverslips before fixation and microscopic analysis.

To estimate the absolute durations of G2 and S phases HCT-116 cells were briefly pulsed with BrdU (10 μM, 15 min) and chased in BrdU-free medium for incremental periods of time from 3 to 11 h (1 h increments) before collection. Cells were then fixed in paraformaldehyde and immunostained for BrdU as described herein. After staining of DNA with DAPI (0.5 μg/mL) cell populations were scored for the presence of BrdU-positive mitotic cells under the fluorescence microscope (Olympus BX50). G2 length was estimated as the shorter chasing time that resulted in ≈50% of BrdU-labeled mitotic cells, and the duration of S phase as the interval of time during which ≥ 50% of mitotic cells displayed staining for BrdU [13, 27].

Duration of the G1 plus G2 phases of the cell cycle was assessed using a cumulative (or saturation) labeling method [32]. Briefly, exponentially growing HCT-116 cells were continuously pulsed with BrdU (10 μM) for incremental periods from 1.5 h to 9 h (1.5 h increments) before collection. Cells were fixed in paraformaldehyde and immunostained for BrdU, and the percentage of BrdU-positive cells was scored for each time point. The duration of G1 + G2 stages was estimated as the minimum pulsing time required for ≈100% of the cells to become positive for BrdU [33].

To judge the absolute length of S phase we also used the so-called “leaving fraction” method [28, 29]. To this end, HCT-116 cells were first pulsed with BrdU (10 μM, 15 min) and chased in BrdU-free medium for 75 min before exposure to a second pulse with EdU (15 μM, 15 min). Cells were then processed for the simultaneous detection of BrdU and EdU, as described here. The leaving fraction, corresponding to the fraction of cells that although initially in S phase – and thus BrdU-positive - have left S phase during the chasing period (thus EdU-negative) was used for extrapolation of the length of S phase according to the formula: *BrdU^positive^/BrdU^positive^ + EdU^negative^ X chase time (h) = Length of S (h)*.

### Cell cycle synchronization

In order to synchronize exponentially growing HCT116 cells at G1/S phase transition, we used the double-thymidine block approach. Briefly, cells were incubated in culture medium containing thymidine at a final concentration of 2 mM for 12 h (1^st^ thymidine block), allowing time for cells to arrest in S phase. Thymidine was removed through repeated washes with fresh medium and cells were incubated with fresh thymidine-free medium for 7.5 h, to allow full exit from S phase. Cells were subsequently incubated with 2 mM thymidine for another 12 h (2nd thymidine block) to obtain a population precisely arrested at the G1/S phase transition that will progress into S phase upon release from thymidine.

### Metaphase spreads

Metaphase spreads were prepared as described [43]. EdU-substituted DNA was detected using the Click-iT assay exactly as described herein for flow cytometry except that the 100 μL of reaction buffer were applied per coverslip (4 cm^2^). Total DNA was stained with DAPI (0.5 μg/mL) immediately before mounting in Vectashield and imaging by confocal microscopy.

### Western blotting

For immunoblotting, cell lysates prepared in boiling 1X Laemmli´s sample buffer were supplemented with PMSF (1 mM) and a commercially available mixture of protease inhibitors (Complete Mini EDTA-free; Roche Diagnostics, Mannheim, Germany; 1 tablet/mL). DNA was first fragmented mechanically by passing the sample into a syringe (≈10 times) through a 25-gauge needle and, subsequently, after supplementation with MgCl_2_ (5 mM), by digestion with benzonase (0.4 Units/ml; Sigma-Aldrich) for 30 min at room temperature. Lysates were then separated on 12 or 14% SDS-PAGE under reducing conditions and transferred to nitrocellulose membranes (Schleicher & Schuel, Keene, NH). Membranes were blocked for 1 h with 5% nonfat dry milk powder in PBS and incubated for a minimum of 2 h with the specific primary and secondary antibodies. Antibodies used for immunoblotting were diluted in PBS supplemented with nonfat dry milk (2.5%) and Triton X-100 (0.05%) and used at the following dilutions: anti-phospho-RPA32/RPA2 (Ser4/Ser8; 1/2000), anti-RPA32/RPA2 (total RPA32; 1/1000), anti-phospho-histone H2A.X (Ser139; 1/1000), anti-histone H2A.X (total H2A.X; 1/1000), and peroxidase-conjugated affinity-purified goat anti-mouse and goat anti-rabbit were diluted at 1/3000. Total H2A.X provided loading controls. The detection reaction was developed by enhanced chemoluminescent (ECL) staining according to the specifications of the manufacturer (ECL Amersham, Western Blotting Detection Reagents, UK).

### Alkaline comet assay

DNA strand breaks were measured using Trevigen Comet Assay kit (Trevigen Inc., Gaithersburg, MD, USA). Cells were resuspended in ice cold PBS (Ca^2+^ and Mg^2+^ free) to a concentration of 1 × 10^5^ cells/ml. A 5 μl aliquot of cells was added to 50 μl of molten 1% low-melting agarose warmed to 37 °C. 50 μl were immediately pipetted and evenly spread onto the comet slides. Slides were incubated at 4°C in the dark for 10 min to accelerate gelling of the agarose disc and then transferred to prechilled lysis solution (2.5 M NaCl, 100 mM EDTA, 10 mM Tris-base, 1% sodium lauryl sarcosinate, 1% Triton X-100, pH 10) for 30 min at 4°C. A denaturation step was performed in alkali solution (300 mM NaOH, 1 mM EDTA, pH > 13) at room temperature for 30 min in the dark. Slides were then transferred to prechilled alkaline electrophoresis solution pH > 13 (300 mM NaOH, 1 mM EDTA) and subjected to electrophoresis at 1 V/cm, 300 mA for 30 min in the dark at 4°C. Subsequently, the slides were washed with deionized water and immersed in 70% ethanol at room temperature for 5 min and air dried. DNA was stained with 100 μl of SYBR Green I dye (supplied with the kit) for 10 min at 4°C in the dark and immediately analyzed using a CCD camera (Roper Scientific Coolsnap HQ CCD, Roper Technologies Inc., Sarasota, FA, USA) attached to a Zeiss Axiovert 200 M wide field fluorescence microscope. For each slide, 100 randomly chosen comets were analyzed with an excitation filter of 450–490 nm and an emission filter of 515 nm. Images were scored for tail length and percentage of DNA in tail using the Tritek CometScore Freeware v1.5 image analysis software (TriTek Corp., Sumerduck, VA, USA).

### Statistical analysis

Data are reported as the mean ± SD. Results were compared by 2-tailed Student's *t* test for two groups and one-way ANOVA followed by Dunnett's multiple comparison test for multiple groups. GraphPad Prism version 5.03 for Windows (GraphPad Software, La Jolla, CA, USA) was used for statistical analysis. Differences were considered statistically significant at *P* < 0.05.

When using incremental pulsing times with EdU in the context of EdU -Coupled-Fluorescence-Intensity analysis (E-CFI), the duration of S phase was estimated as the first time point after which maximal EdU-coupled fluorescence intensities clustered within 2 SDs from each other. Also in the context of E-CFI, total length of cell cycle was estimated from the lengths of G1, G2 and S phases by standard error propagation.

## SUPPLEMENTARY MATERIALS FIGURES AND TABLES


