# Supplementary material for: Quantification of cell cycle kinetics by EdU (5-ethynyl-2′-deoxyuridine)-coupled-fluorescence-intensity analysis
Source: Oncotarget. 2017 Apr 15;8(25):40514–32. doi: 10.18632/oncotarget.17121 (PMC5522303; doi:10.18632/oncotarget.17121)
Supplement: Supplementary file 1 [file oncotarget-08-40514-s001.pdf]

Quantification of cell cycle kinetics by EdU (5-ethynyl-2'-deoxyuridine)-coupled-fluorescence-intensity analysis

Supplementary Materials

Supplementary Table 1: Comparison of estimates for cell cycle phase length calculated for HCT-116 DNA-PK WT (DAPI stained, 3 independent experiments, mean + SD) using two different cell cycle analysis algorithms

Cell Cycle Algorithm	G1	G2	S
**Dean Jett Fox**			
Population percentage	32.3 ± 5 %	25.9 ± 4.2 %	33.5 ± 8.3 %
Estimated length*	4.8 ± 0.7 h	3.9 ± 0.6 h	5 ± 1.2 h
**Watson Pragmatic**			
Population percentage	28.9 ± 4.3 %	27.5 ± 16.5 %	44.9 ± 7.9 %
Estimated length*	4.3 ± 0.6 h	4.1 ± 2.5 h	6.7 ± 1. 2 h

*Estimated cell phase durations are derived from the percentage of cells in each cell cycle stage calculated using cell cycle algorithms within FlowJo software, assuming a total cell cycle length of 15 h.

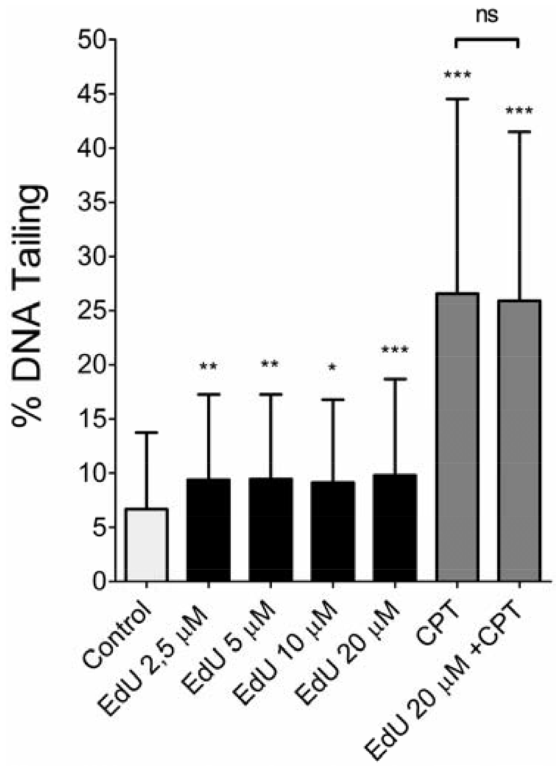

Supplementary Figure 1: DNA damage induced by EdU–comet assay. HCT-116 cells exposed for 11 h to EdU (2.5, 5, 10 and 20 µM; negative controls exposed to DMSO) or CPT (positive control; +/- EdU 20 µM) and analyzed by alkaline single-cell gel electrophoresis (comet assay; parameter: percentage of DNA in tail). Results are presented as mean + SD; ***p < 0.001; **p < 0.01; *p < 0.05; ns: p > 0.05.

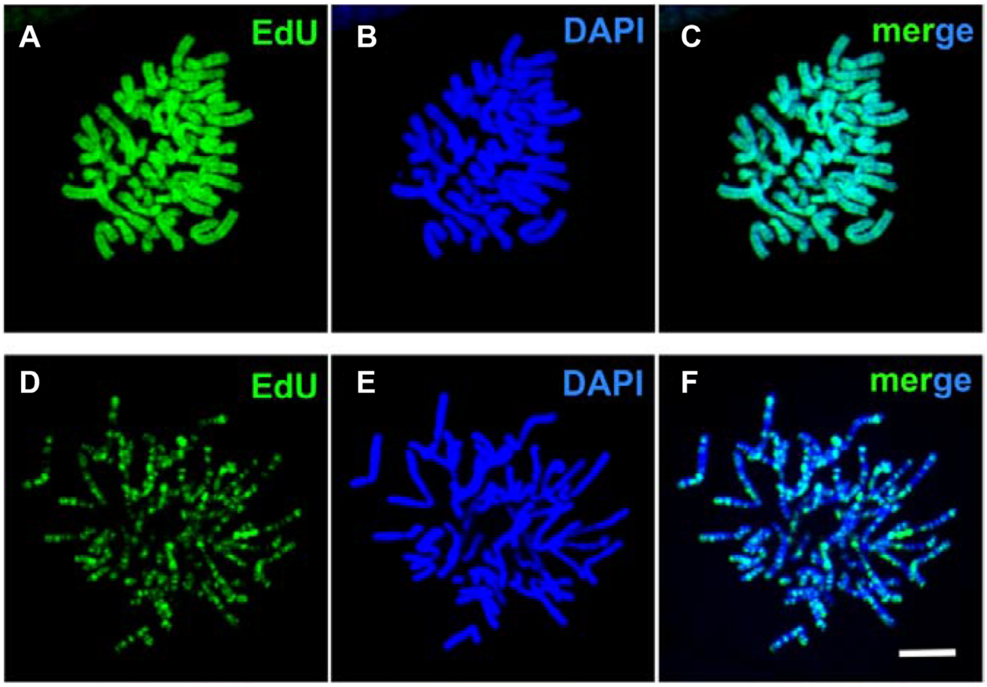

Supplementary Figure 2: Metaphase spreads after full and partial S phase labeling. (**A–C**) Synchronized HCT-116 cells (double-thymidine block) were pulsed with EdU (10 µM; 7 h) for a full S phase before harvesting in M phase. Metaphase spreads were stained for (a) EdU (Click-iT; Alexa 488; green) and (b) DNA (DAPI; blue); (c) merge of EdU and DAPI staining. Note labeling for EdU across the entire length of chromosome arms. (**D–F**) HCT-116 cells synchronized as above were allowed to progress 4 h into S phase and briefly pulsed with EdU (15min, 10 µM) before harvesting in M phase. Metaphase spreads were stained for (d) EdU and (e) DNA, as above; (f) merge of both channels. Note discontinuous (banded) labeling for EdU. Bar: 10 µm.

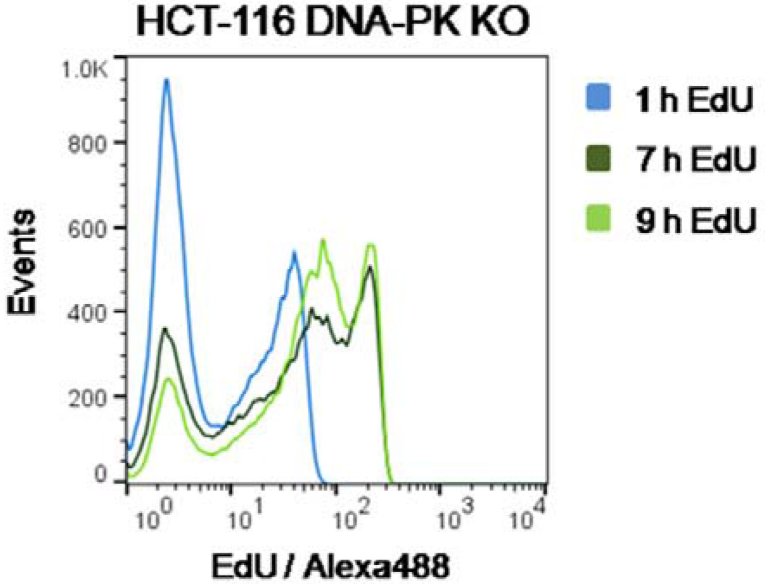

Supplementary Figure 3: Estimation of S phase length in HCT-116 DNA-PK KO cells using E-CFI. Asynchronous HCT-116 DNA-PK KO cells cells were exposed to EdU (10 µM) for 1 to 11 h (1 h increments) followed by detection of EdU-DNA using Click-iT chemistry (Alexa 488) and analysis by flow cytometry. Fluorescence intensities after 1 h (blue), 7 h (dark green) and 9 h (light green) of EdU incorporation are depicted along the x axis in logarithmic scale (other pulsing times not shown). Note that the 7 h and 9 h time points share identical maxima of EdU-coupled fluorescence intensity.

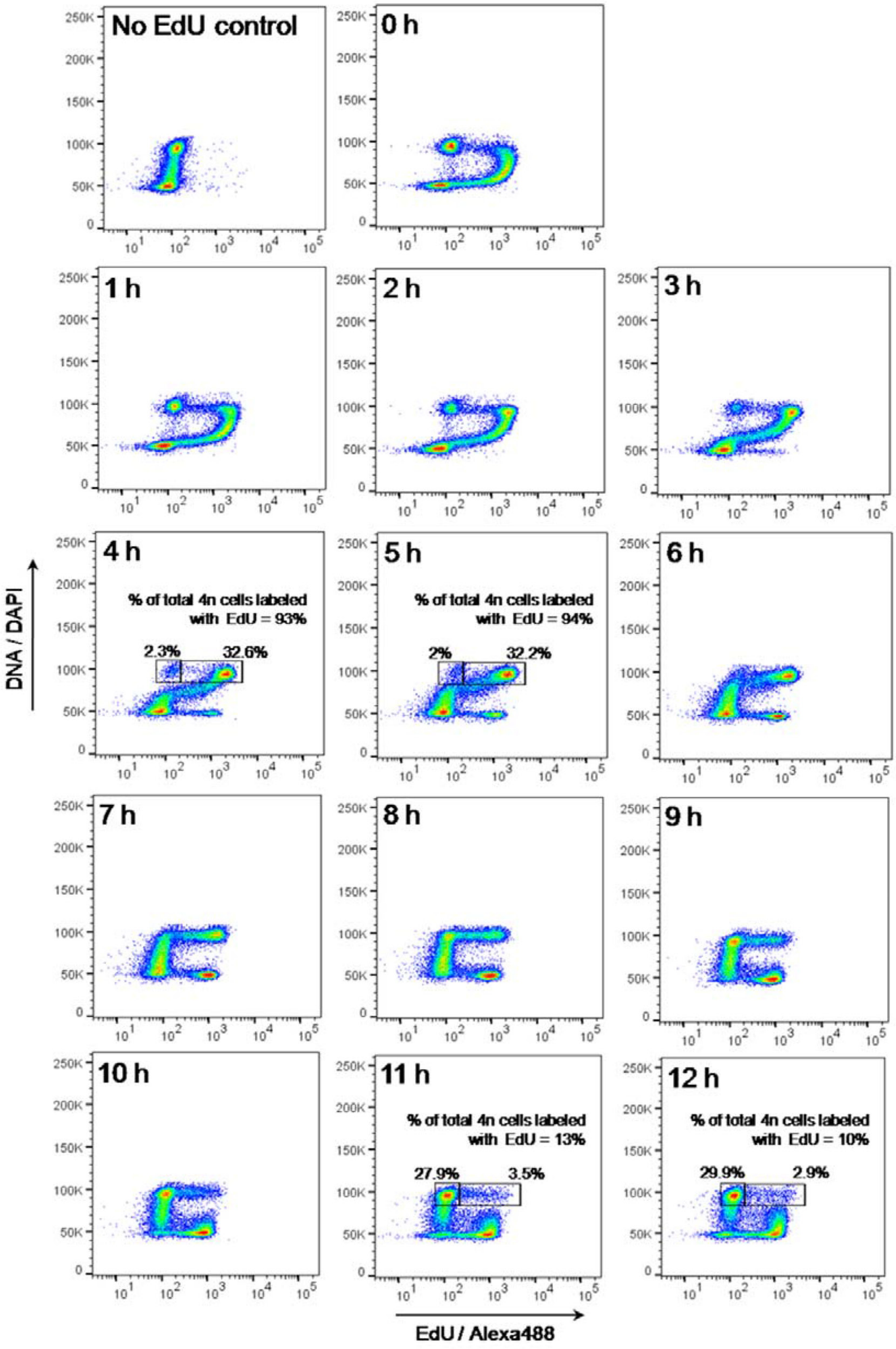

Supplementary Figure 4: Dual parameter histograms (EdU vs total DNA) after EdU pulse-chase. Asynchronous HCT-116 cells pulsed with EdU (10 µM; 30 min) were either collected immediately after pulsing (0 h) or else chased for the indicated times (1–12 h) before collection. EdU-DNA was detected using Click-iT chemistry (azide-Alexa 488); total DNA was stained with DAPI before dual-parameter processing (EdU *vs* total DNA). Controls comprise EdU-minus cells that were exposed to azide-Alexa 488 (EdU detection system) before staining with DAPI (No EdU control). Selected events (within boxes) in chase times 4 and 5 h, and 11 and 12 h denote populations with 4n DNA that are either EdU-negative (left) or EdU-positive (right). The corresponding percentages within the whole cell population are denoted. Also denoted are the percentages of 4n DNA cells that are EdU-positive within the whole 4n DNA population. Data from a single experiment are shown.

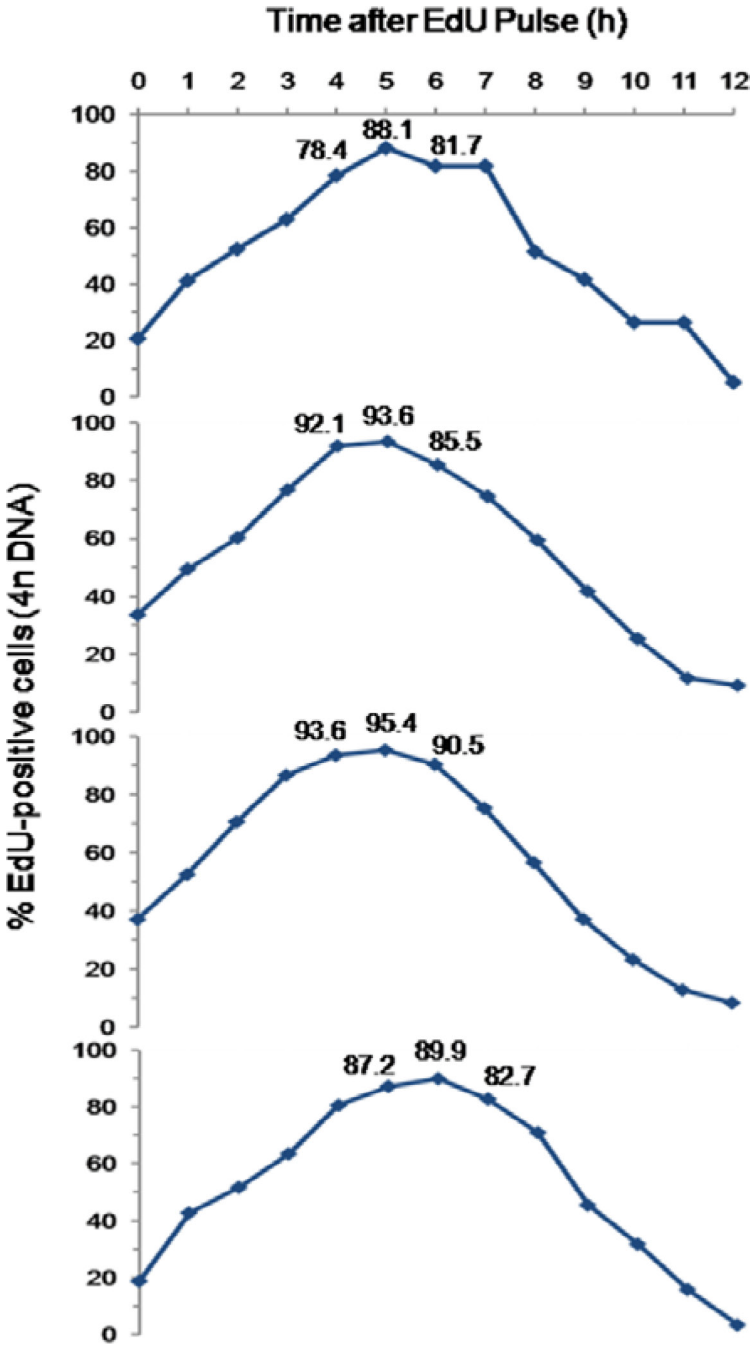

Supplementary Figure 5: Fraction of EdU-labeled cells harboring 4n DNA over time – single experiments. Asynchronous HCT-116 cells pulsed with EdU (10 µM; 30 min) were collected immediately after pulsing (0 h) and for the indicated times (1–12 h) thereafter. Shown at defined times after EdU pulsing are the percentages of cells with 4n DNA content that are labeled for EdU; numbers above the curves denote the highest percentages of EdU-positive cells. Data were extracted from dual-parameter histograms after detection of EdU-DNA using Click-iT chemistry (azide-Alexa 488) and staining total DNA with PI. Data shown are from four independent experiments.

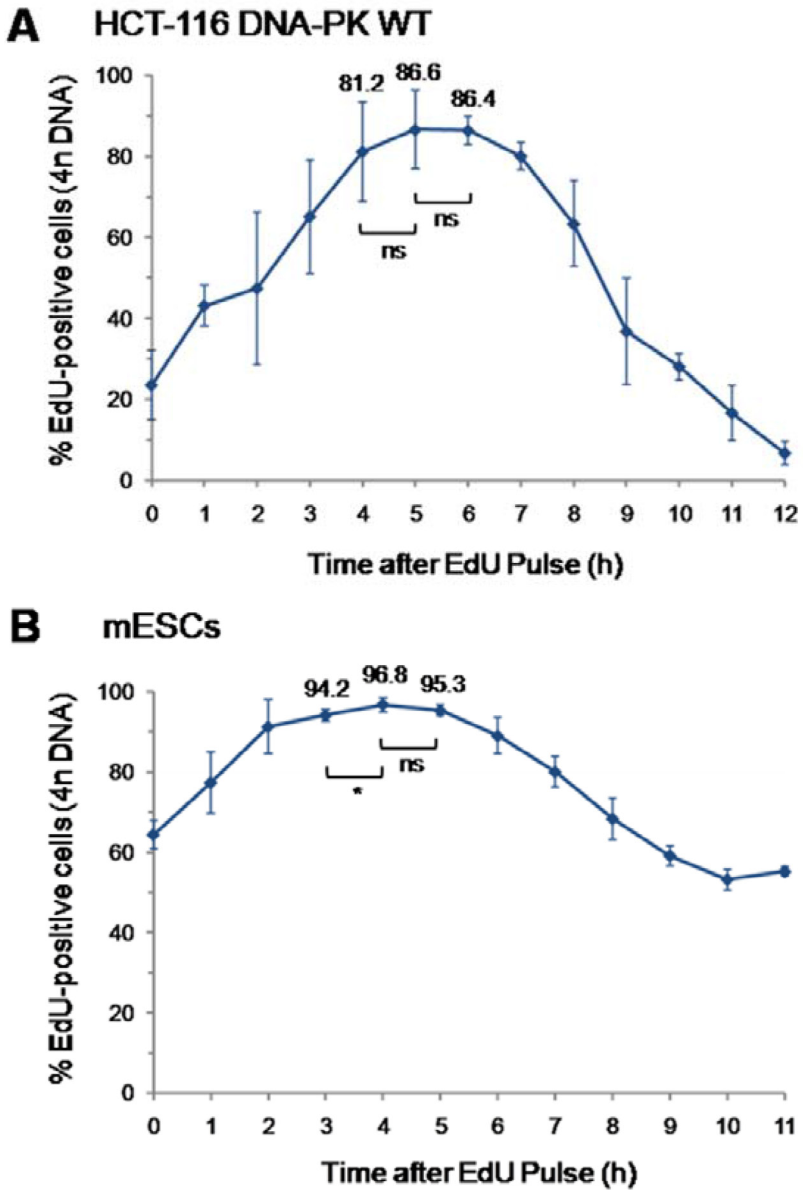

Supplementary Figure 6: Fraction of EdU-labeled cells harboring 4n DNA over time – pooled data. (**A**) Asynchronous HCT-116 cells were pulsed with EdU (10 µM; 30 min) and collected at the indicated times (0–12 h). Dual-parameter histograms (EdU/DNA *vs* total DNA/PI) were used to distinguish cells with 4n DNA content that incorporated EdU. Represented over time are the percentages of cells with 4n DNA content that are EdU-positive. (**B**) mESCs were pulsed with EdU (5 µM; 30 min) and processed as above. Results are presented as mean + SD from four independent experiments per cell line. Values for maximal percentages are presented above time points. 2-tailed Student's t test was used to compare neighboring time points; $*p < 0.05$; ns: $p > 0.05$.
